# A single residue substitution accounts for the significant difference in thermostability between two isoforms of human cytosolic creatine kinase

**DOI:** 10.1038/srep21191

**Published:** 2016-02-16

**Authors:** Huihui Liu, Yan-Song Gao, Xiang-Jun Chen, Zhe Chen, Hai-Meng Zhou, Yong-Bin Yan, Haipeng Gong

**Affiliations:** 1 MOE Key Laboratory of Bioinformatics, School of Life Sciences, Tsinghua University, Beijing 100084, China; 2 State Key Laboratory of Membrane Biology, School of Life Sciences, Tsinghua University, Beijing 100084, China; 3 Zhejiang Provincial Key Laboratory of Applied Enzymology, Yangtze Delta Region Institute of Tsinghua University, Jiaxing 314006, China

## Abstract

Creatine kinase (CK) helps maintain homeostasis of intracellular ATP level by catalyzing the reversible phosphotransfer between ATP and phosphocreatine. In humans, there are two cytosolic CK isoforms, the muscle-type (M) and the brain-type (B), which frequently function as homodimers (hMMCK and hBBCK). Interestingly, these isoenzymes exhibit significantly different thermostabilities, despite high similarity in amino acid sequences and tertiary structures. In order to investigate the mechanism of this phenomenon, in this work, we first used domain swapping and site-directed mutagenesis to search for the key residues responsible for the isoenzyme-specific thermostability. Strikingly, the difference in thermostability was found to principally arise from one single residue substitution at position 36 (Pro in hBBCK vs. Leu in hMMCK). We then engaged the molecular dynamics simulations to study the molecular mechanism. The calculations imply that the P36L substitution introduces additional local interactions around residue 36 and thus further stabilizes the dimer interface through a complex interaction network, which rationalizes the observation that hMMCK is more resistant to thermal inactivation than hBBCK. We finally confirmed this molecular explanation through thermal inactivation assays on Asp36 mutants that were proposed to devastate the local interactions and thus the dimer associations in both isoenzymes.

Creatine kinase (CK, EC 2.7.3.2) belongs to the phosphagen kinase superfamily, and functions as an “energy reservoir” to connect the production and consumption of ATPs in different cellular compartments^1^,^2^. Historically, CK has been taken as a model macromolecule for the mechanistic studies on protein folding. Through chemical and/or thermal denaturation, the mechanisms of inactivation and multistate folding of CK have been investigated extensively, using numerous biophysical methods including ultraviolet absorption, fluorescence, infrared spectrum, circular dichroism and sedimentation velocity^3^,^4^,^5^,^6^. In addition, the presence of several isoforms of CK in various tissues and/or subcellular organelles enables the study of molecular evolution and environmental adaptation using this enzyme^7^,^8^.

Although present in many chordates and invertebrates, CK is the only phosphagen kinase in vertebrates^9^. Up to date, four CK isoenzymes have been identified in higher vertebrates, among which the two cytosolic isoforms, the muscle type (MCK) and the brain type (BCK), can combine to form either homodimer (MMCK and BBCK) or heterodimer (MBCK)^10^, while the two mitochondrial isoforms (the ubiquitous type and the sarcomeric type) may assemble as either octamer or dimer depending on environmental conditions^11^. The relatively low sequence identity between the cytosolic and mitochondrial CK isoforms of ~60–65% suggests their early divergence in phylogeny^12^,^13^. The two cytosolic CK genes are proposed to evolve late from the same ancestor through gene duplication^14^, and accordingly they exhibit a high sequence identity of 80%.

Despite the high sequence similarity, MMCK and BBCK are quite dissimilar in their tissue distributions, intracellular binding partners, catalytic efficiencies and stabilities^7^,^15^,^16^,^17^. Mainly distributed in muscle and heart, MMCK can be recruited to the sarcomere and is functionally coupled to myosin via myomesin or MyBPC^18^,^19^,^20^. BBCK, which is broadly expressed in many tissues, especially in brain, retina and some glands, can activate neuron-specific K^+^-Cl^−^ co-transporter KCC2^21^ and can affect the spreading and migration performance of astrocytes and fibroblasts^22^. Importantly, inactivation of BBCK by oxidation in the cytoplasm of human brain cells is associated with numerous neurodegenerative disorders including Alzheimer’s disease and Pick’s disease^23^,^24^. At the normal body temperature, the human BBCK (hBBCK) presents higher catalytic power as well as much lower thermal stability than the human MMCK (hMMCK)^7^. These distinct properties between isoforms make cytosolic CK a potential candidate to study the molecular evolution and adaptation of isoenzymes to various environmental conditions.

According to the available crystal structures, the two human cytosolic CK isoforms (hBBCK and hMMCK) adopt highly similar tertiary structures^16^,^25^, with each subunit (MCK or BCK) composed of a small N-terminal domain (NTD), a large C-terminal domain (CTD) and a long linker to connect them. Notably, the active site is located in the cleft between NTD and CTD^16^,^25^. As shown in Fig. S1, the two dimeric isoenzymes share a nearly identical core structure with minor structural deviations located at the N- and C-termini as well as some intervening loops^16^. Despite the high structural similarity, hMMCK is much more stable (by ~15 °C) than hBBCK during heat denaturation. Moreover, the two isoenzymes are inactivated in distinct manners upon heating^7^. Although both of them experience dimer dissociation^7^,^26^, the thermal inactivation is irreversible for hMMCK, but is highly reversible for hBBCK at temperatures below 55 °C^7^.

Among the factors proposed in previous studies as contributors to the hyperthermostability of thermophilic enzymes^27^,^28^,^29^,^30^, the number of ion pairs is conventionally considered as the most important one, since it is the only indicator that statistically significantly rises in the thermophilic enzymes when compared to their mesophilic homologues^31^. This factor, however, does not show significant difference between the hBBCK and hMMCK isoenzymes, and thus fails to explain the drastic distinction in their thermal stabilities. Therefore, it is of great interest to investigate the factors determining the isoenzyme-specific thermostability of human cytosolic CKs.

Considering the high sequence identity and structural similarity between hBBCK and hMMCK, their significant difference in thermal stabilities should arise from the few amino acids that are distinct in the two isoenzymes. In this work, we first engaged mutation analysis and biophysical examination to pinpoint these key residues. Using a strategy combining domain swapping and site-directed mutagenesis, the residue 36 (Pro in hBBCK vs. Leu in hMMCK) was identified as the most important one responsible for the isoenzyme-specific thermostability of human cytosolic CKs. We then conducted molecular dynamics (MD) simulations to investigate the molecular mechanism of the above phenomenon at the atomistic level. Based on the simulations, when Leu36 is replaced by Pro36 in both isoenzymes, the binding affinity at the dimer interface is greatly weakened, which facilitates the dimer dissociation. Subsequent analysis on hBBCK implies that the alteration at the dimer interface may originate from the propagation of local perturbation around the residue 36 through an interaction network, and that the additional local interaction formed around Leu36 stabilizes the dimer interface by suppressing the movement mode of dimer dissociation. This molecular explanation was finally confirmed by a control experiment in both hBBCK and hMMCK, where Asp was introduced at position 36 to completely abolish the local interactions and to greatly impair the thermostability.

## Results

### The region determining the isoenzyme-specific thermostability was narrowed down by domain swapping

**Figure 1:**
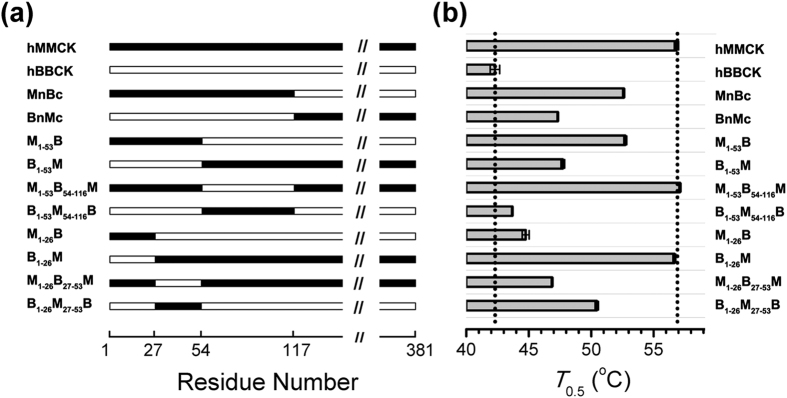
Identification of amino acid segment responsible for the isoenzyme-specific thermostability. **(a)** Schematic representation of the CK chimeras constructed. **(b)** Semi-inactivation temperatures (*T*_0.5_) for the WT CK and the chimeras. The original thermal inactivation curves are shown in supplemental Fig. S2a.

To identify the amino acid residues contributing to the isoenzyme-specific thermostability, chimeras were firstly constructed by exchanging NTDs (residues 1–116) of the two isoenzymes (MnBc and BnMc in Fig. 1a). The hMMCK is 14.5 °C more stable than the hBBCK, while the thermal stabilities of the chimeras fall between the two wild-type (WT) isoforms (Figs 1b & S2a; Table S1). Particularly, replacing the NTD of hBBCK by that of hMMCK (MnBc) raises the midpoint temperature of inactivation (*T*_0.5_) by 10.3 °C. In contrast, the *T*_0.5_ only elevates by 5 °C when similarly substituting the CTD of hBBCK by that of hMMCK (BnMc) (Fig. 1b). These observations suggest the larger contribution of NTD to the isoenzyme-specific thermostability of human cytosolic CKs.

To further identify the key residues within the NTD, the NTD was subdivided into two regions, residues 1–53 and residues 54–116. Four chimeras, denoted as B_1–53_M, M_1–53_B_54–116_M, M_1–53_B and B_1–53_M_54–116_B respectively, were constructed, where B and M denote the sources of amino acid sequences and the subscripts indicate the ranges of residues (Fig. 1a). The thermal inactivation curve of M_1–53_B_54–116_M is almost indistinguishable from that of WT hMMCK (Fig. S2a), with nearly identical *T*_0.5_ values (Fig. 1b and Table S1). Similarly, B_1–53_M_54–116_B and WT hBBCK show overlapping thermal inactivation curves (Fig. S2a) and close *T*_0.5_ values (Fig. 1b and Table S1). These results negate the significant contribution of residues 54–116 in the isoenzyme-specific thermostability. Meanwhile, the thermal inactivation curves and *T*_0.5_ values for the chimeras with residues 1–53 swapped (B_1–53_M and M_1–53_B) resemble those of the chimeras with NTD swapped (BnMc and MnBc) respectively (Fig. 1b & S2a and Table S1), which supports the importance of residues 1–53 within the NTD.

A subdivision of the region of residues 1–53 was performed to further narrow down the search (Fig. 1a). As shown in Figs 1b & S2a and Table S1, swapping of the N-terminal 26 residues (M_1–26_B and B_1–26_M) does not significantly alter the thermal stabilities of either hBBCK or hMMCK. In contrast, the swapping of residues 27–53 (B_1–26_M_27–53_B and M_1–26_B_27–53_M) restores the thermal inactivation behaviors of MnBc and BnMc. Therefore, the region of residues 27–53 is mainly responsible for the difference in thermal stabilities between hBBCK and hMMCK.

Despite the dominant role of residues 27–53 in thermal stability, this region is less involved in the isoenzyme-specific catalysis of CK. As shown in Table S1, swapping of this region only slightly alters the relative activities of the WT isoforms, while exchanging the CTDs nearly switches the catalytic powers of hBBCK and hMMCK. Therefore, the isoenzyme-specific thermostability and catalytic power of human cytosolic CKs may arise from the sequence differences in the NTDs and CTDs respectively.

### Residue 36 was finally identified to be responsible for the isoenzyme-specific thermostability

**Figure 2:**
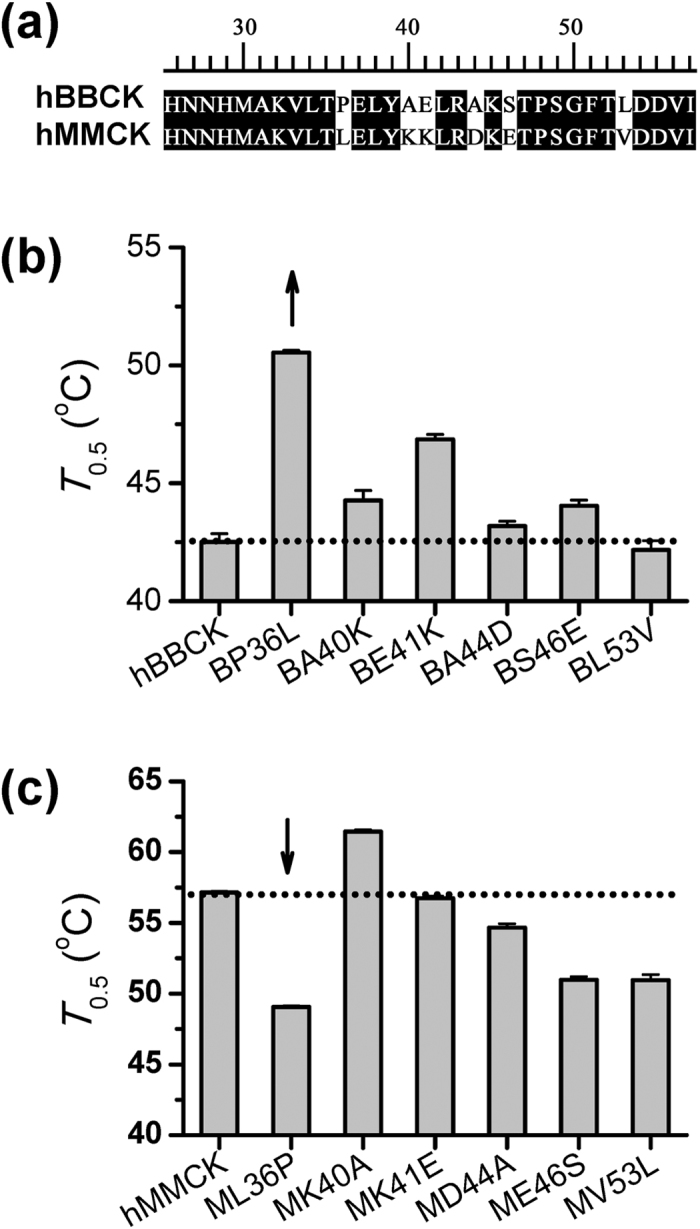
Identification of the key residues responsible for the isoenzyme-specific thermostability. **(a)** Sequence alignment of hBBCK and hMMCK. The identical residues are shown in white and shaded in black. **(b)** Semi-inactivation temperatures (*T*_0.5_) for hBBCK and its mutants. **(c)** Semi-inactivation temperature (*T*_0.5_) for hMMCK and its mutants. The original thermal inactivation curves are shown in supplemental Figs S2b & S2c.

Within the key region (residues 27–53) determining the isoenzyme-specific thermostability as identified above, only 6 out of 27 amino acid residues are different between hBBCK and hMMCK (Fig. 2a). To evaluate the contributions of these residues, site-directed mutagenesis was performed at each of the 6 positions, to mutate the hBBCK and hMMCK toward each other. Most mutations have mild effects on CK activity at room temperature except the K41E and V53L mutants in the hMMCK group (Table S2). The former raises the relative activity by 41% while the latter reduces the catalytic power by 56%.

According to the thermal inactivation curves and *T*_0.5_ values of the mutants summarized in Figs 2b & S2b and Table S2, the P36L and E41K mutations affect the thermal stability of hBBCK the most significantly, raising the *T*_0.5_ values by 8.2 °C and 4.6 °C respectively. In contrast, the L53V mutation does not change the thermal stability at all, while A40K, A44D and S46E only slightly stabilize hBBCK (with 1–2 °C increase in the *T*_0.5_ value). On the other hand, as shown in Figs 2c & S2c and Table S2, the thermal stability of hMMCK is impaired by 7.7 °C, 2.1 °C, 5.8 °C and 5.8 °C in the L36P, D44A, E46S and V53L mutants respectively, but is not significantly affected by the K41E mutation. Surprisingly, the *T*_0.5_ value of hMMCK rises by 4.7 °C in the K40A mutant. Nevertheless, the thermal stabilities of both hBBCK and hMMCK are altered to the highest degree by the mutual mutation at residue 36 (Fig. 2), which suggests the largest contribution of this residue to the isoenzyme-specific thermostability of human cytosolic CKs.

### Residue 36 modulates the isoenzyme-specific thermostability by affecting the dimer dissociation

Our previous work^7^ reported that the thermal inactivation of both hBBCK and hMMCK is accompanied by dimer dissociation and that the relative activity is directly proportional to the content of native dimeric enzymes. Thus, the molecular mechanism of thermal inactivation for the human cytosolic CKs is supposed to be related to the subunit dissociation at the dimeric interface.

To find out whether residue 36 contributes to the isoenzyme-specific thermostability of human cytosolic CKs by affecting dimer dissociation, we conducted conventional MD (cMD) simulations on the four proteins including hBBCK, hMMCK and the corresponding mutants at residue 36 (denoted as BP36L and ML36P respectively) (Table S3). Although the timescale of dimer dissociation process may exceed the observation limit of typical MD simulations, the binding affinities between the subunit pairs can be calculated to quantitatively evaluate the tendency of dimer dissociation. The BP36L and ML36P proteins were constructed from the crystal structures of hBBCK and hMMCK respectively, through point mutation. Following 30 ns pre-equilibrations, all four target proteins are structurally stable during the 100 ns productive simulations (Fig. S3). The binding free energies between the subunit pairs were calculated using the Molecular Mechanics/Poisson-Boltzmann Surface Area (MM/PBSA) method, with entropies omitted considering the similar structures among all proteins. During the 100 ns simulation, the binding free energy of hBBCK converges to a mean value of about −181 kcal/mol, significantly weaker than the mean value of about −202 kcal/mol in BP36L with a p-value <1 × 10^−7^ (Table 1). Therefore, the dimeric interaction in hBBCK becomes stabilized by the P36L mutation, consistent with the rise of *T*_0.5_ value in BP36L (Fig. 2b). Notably, the magnitude of the difference in binding free energies (~21 kcal/mol) is less important, considering the uncertainty in the free energy estimation due to the limitation of the MM/PBSA method. Meanwhile, the binding free energy of hMMCK is significantly weakened from about −192 kcal/mol to about −165 kcal/mol with a p-value <1 × 10^−7^ (Table 1) by the reverse L36P mutation, which agrees with the reduced *T*_0.5_ value in ML36P (Fig. 2c). Notably, the binding free energies between the hBBCK and hMMCK systems cannot be compared directly, both because the hMMCK crystal structure lack the N-terminal residues that are present at the dimeric interface in the hBBCK crystal structure, and because the two systems have non-identical residues at the dimeric interface. The above free energy calculations support our proposition that the residue 36 determines the isoenzyme-specific thermostability of human cytosolic CKs by affecting dimer dissociation.

Subsequently, the binding free energy was further decomposed into the contributions from single residues. The key residues that particularly stabilize the mutant over WT in the hBBCK system were then selected based on the difference in binding free energies between BP36L and hBBCK. The top 18 residues with ΔΔG < −1 kcal/mol are listed in the descending order in Table S4. Similarly, the top residues that particularly destabilize ML36P over hMMCK were also selected based on the difference in binding free energies (ΔΔG > 1 kcal/mol). The two sets of residues identified above are proposed to make significant contributions in stabilizing the dimer interaction in the hBBCK and hMMCK systems respectively, when Pro36 is mutated to Leu36. Interestingly, all of these residues are distributed on the dimer interface (Fig. S4), and many of them are charged residues (Table S4). We finally chose 7 common residues in the two sets (Arg209, Asp55, Asp62, Glu19, Lys156, Arg148 and Asp210) as the key interface residues that account for the stabilization on dimer interaction by the P36L mutation. As expected, all of these 7 charged residues form salt bridges and/or hydrogen bonds with partners in the opposite subunit to stabilize the interface (Fig. S5 and Table S5).

To validate the above *in silico* simulations and calculations, we performed *in vitro* gel filtration assays to check the compositions of the four proteins (hBBCK, hMMCK, BP36L and ML36P) after thermal inactivation (Fig. S6). Similar to the results reported previously^7^, hBBCK becomes partially dissociated into monomers at 45 °C, while no monomeric intermediate could be detected for hMMCK during thermal inactivation. Moreover, BP36L maintains the dimeric structure well at 45 °C, while stable monomeric form is detected for ML36P at 52 °C. The relatively low peak intensity in ML36P is caused by severe aggregation. In summary, the observations in the gel filtration experiments are consistent with the free energy calculations, both showing that the dimer dissociation is substantially suppressed when Pro36 is mutated to Leu36 in both the hBBCK and hMMCK systems.

### The dimer dissociation can be inhibited by suppressing the movement amplitude of interface residues

Although the binding affinity between subunits at the dimer interface could be evaluated through the MM/PBSA calculation in the previous section, large-scale conformational changes such as the dimer dissociation cannot be directly observed in cMD simulations due to the limited simulation time. However, the tendency of large-scale collective movements could be identified by decomposing the simulation trajectories to mutually orthogonal movement modes using the principal component analysis (PCA). The collective movement of interest is frequently distributed over several principal component (PC) modes derived from the PCA analysis. In this work, we adopted the functional mode analysis (FMA) developed by de Groot and colleagues^32^,^33^ to identify the collective movements related with dimer dissociation. The FMA technique can find a linear combination of PC modes (called functional mode) that is maximally correlated with a self-defined biological function. Here, we used the total solvent accessible surface area (SASA) of all interface residues as the biological function to quantify the degree of dimer dissociation. In specific, the interface residues are defined from the static crystal structure, as the residues whose SASA values become reduced during the association of the two rigid subunit structures.

The functional mode derived from the FMA analysis could be evaluated by the Pearson correlation coefficient (PCC) between the projection along the computed mode and dimer dissociation, the latter of which is evaluated by the total SASA of interface residues. Generally, a larger absolute PCC value indicates a stronger tendency of dimer dissociation. As shown in Table S6, hBBCK exhibits the strongest correlation (PCC = 0.690), followed by ML36P (PCC = 0.543), BP36L (PCC = −0.437) and hMMCK (PCC = −0.286). This order agrees with the thermal inactivation experiment, thereby further supporting our proposition that thermal stability of human cytosolic CKs is principally disrupted through dimer dissociation. We selected the functional mode of the WT hBBCK (with the highest absolute PCC) as the collective movement that best reflects the dimer dissociation in human cytosolic CKs. However, the weak mutual correlations between the functional modes identified from the four proteins negate the possibility to investigate this selected best mode in all systems. As a compensation, this best mode was then projected on the top 20 PC modes of all four proteins to detect the best PC mode to describe dimer dissociation within each respective system. As shown in Fig. S7a, the second PC modes (PC2) in both WT and mutant of the hBBCK trajectories show considerable agreement with the collective movement of dimer dissociation (0.90 for hBBCK and 0.55 for BP36L respectively in terms of absolute PCC values). In addition, the two PC2 modes show mutual agreement (absolute PCC = 0.66), implying the possibility to use them to compare the behaviors of dimer dissociation between the mutant and WT in the hBBCK system. In the hMMCK and ML36P trajectories, although the first PC modes (PC1) can partially describe the collective movement of dimer dissociation, the correlations are relatively weak (0.53 for hMMCK and 0.38 for ML36P respectively).

Given the above results, we chose WT and mutant of the hBBCK system for further analysis and compared the residue movements in their best PC modes (PC2) of the two proteins. Although both PC2 modes have been normalized, most residues, especially those in chain A, show smaller amplitudes of movement in BP36L than in hBBCK (Fig. S8a). In Table 2, the moving amplitudes of residue 36 as well as seven key interface residues (identified through free energy calculation in the above section, see Table S4) in the PC2 modes are listed side-by-side for hBBCK and BP36L. All residues show greatly suppressed movement in BP36L than in hBBCK, except for Lys156 in chain B with comparable movement amplitudes in the two proteins. In contrast, the directions of residue movement in the two proteins generally agree with each other (Table 2 and Fig. S8b). In conclusion, the P36L mutation in hBBCK suppresses the movement of most residues, particularly the key interface residues, in the PC mode that best reflects dimer dissociation, implying that Leu36 may stabilize the dimer interface by inhibiting the residue movements that cause subunit dissociation.

### The movement of interface residues is modulated by changing the compactness of residue interaction network

Although the above analysis indicates that the P36L mutation may stabilize the dimer interface by suppressing the dissociation-related movements, residue 36 and the ones located at the interface do not make direct contact, which requires the presence of interaction pathways to connect them. We performed network analysis to identify these interaction pathways. In specific, each residue is represented as a node in the network and two nodes are connected only when the following two criteria are satisfied simultaneously: 1) the two residues show correlated movements in the PC2 mode with the scalar angle between moving directions <45°; 2) the two residues make physical contact, requiring that at least one pair of heavy atoms from two candidate residues approximate to a distance of <4.5 Å in at least 75% of the frames in the overall simulation trajectory. Generally, the network topology is highly dependent on the parameter values chosen for the network construction. Here, we tested our network analysis using a variety of parameter values and obtained consistent results (see *Methods* in the Supplementary Information for details), which supports the robustness of our calculation.

The constructed network of BP36L contains more nodes and edges than that of hBBCK, as justified by the various network properties shown in Table 3, particularly the average node degree and the average clustering coefficient, which evaluate the extent of connections around each node on average. This distinction in the network topology was then corroborated as statistically significant using a bootstrap protocol, by randomly choosing 80% of the frames from each trajectory for network construction and repeating the process for 10 times (Table S7). Therefore, the P36L mutation intensifies the interaction network of the hBBCK system, which intrinsically suppresses the residue movement in the PC mode that best reflects the dimer dissociation.

**Figure 3:**
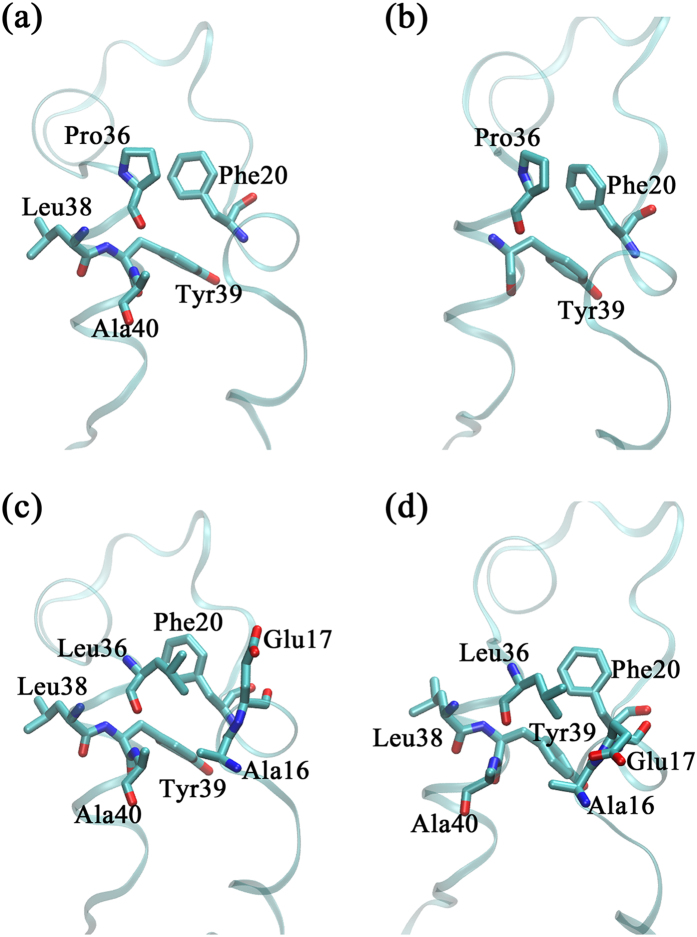
Network connections around residue 36 in **(a)** chain A of hBBCK, **(b)** chain B of hBBCK, **(c)** chain A of BP36L and **(d)** chain B of BP36L. Structures were captured from the last frames of the cMD trajectories. All structural figures were made using VMD 1.9.1^53^.

To further decipher how a single residue substitution causes such changes in the interaction network, we focused on the local network around residue 36. After the mutation from Pro to Leu, residue 36 forms interactions with additional residues, Ala16 and Glu17 (Fig. 3 and Table S8). Such additional local interactions are supposed to strengthen the compactness of the overall network eventually. Moreover, the residue 36 is connected with all key interface residues in both protein networks (Table S9), which enables the remote modulation on the movement of interface residues by the local change at residue 36. Notably, the crosstalk between residue 36 and interface ones is reinforced in BP36L when compared to hBBCK, as indicated by either the reduced length of the shortest path or the raised number of pathways when the length does not change (Table S9). This observation is robust among 10 repeated bootstrap calculations, where 80% of frames were randomly chosen from the trajectory for network construction in each repeat (Table S10). Conclusively, the mutation of Pro to Leu strengthens the remote interaction between residue 36 and the interface ones and therefore helps stabilize the dimer interface by suppressing the movement of interface residues.

The above network analyses indicate that the stabilization of the dimer association in BP36L may arise from the locally intensified residue interaction around Leu36. As shown in Fig. 3, residues connected with the side group of Pro36/Leu36 in the networks are all nonpolar, except for Glu17 that exerts the interaction using its β-carbon atom. Therefore, the hydrophobic side group of Leu36 is supposed to enhance the local interactions, thereby further stabilizing the dimer interface. Theoretically, introduction of an acidic residue at position 36 should devastate all of the above interactions (including Glu17), which supposedly triggers dimer dissociation by disrupting the interaction network. We then performed an *in vitro* validation experiment, by introducing the Asp36 mutation in both hBBCK and hMMCK systems and measuring the thermal inactivation curves. As expected, the thermostability of hBBCK is severely impaired by the mutation, with the *T*_0.5_ dropped by 7 °C in BP36D (Fig. S9). A similar trend is also observed in hMMCK, with the *T*_0.5_ reduced by 12 °C in ML36D (Fig. S9). Therefore, our mechanistic explanation derived from the network analysis on hBBCK is applicable to hMMCK, indicating the validity of our proposition that residue 36 modulates the dimer dissociation (and thermostability) of human cytosolic CKs through an interaction network. Notably, mutation of residue 36 to Asp greatly reduces but cannot completely remove the difference in thermostabilities between the two isoenzymes of human cytosolic CKs (Fig. S9), which indicates the presence of additional factors contributing to the isoenzyme-specific thermostability.

## Discussion

### Recapitulating the molecular model

In this work, we first searched for the residues that are responsible for the isoenzyme-specific thermostability of human cytosolic CKs by changing the sequences of hBBCK and hMMCK toward each other and by examining the effect using thermal inactivation. Through a series of domain swapping and point mutations, we finally identified the residue at position 36 as the key contributor to the difference in thermal stabilities between the two isoforms of human cytosolic CKs. Interestingly, the key residue 36 is located at the N-terminal cap of the third α-helix (α3) in the NTD and is therefore not directly involved in either dimer association or enzymatic catalysis. Although the mutation from Pro to other residues (e.g., Glu, Thr and Ala) was reported to impair the stability of hyperthermophilic adenylate kinase according to both MD simulations and NMR experiments^34^, our study showed a contrary pattern in CK, in that the mutation from Pro to Leu stabilizes the protein.

According to the subsequent MD simulations and gel filtration assays, we found that the residue 36 controls the thermostability by modulating the binding affinity at the dimer interface, although it is located neither at the interface nor within the active site. Further calculations on the hBBCK system indicate that the P36L mutation stabilizes the dimer interface by suppressing the motion amplitudes of interface residues in the global movement mode that best reflects the dimer dissociation. Although the residue 36 does not make physical interactions with the interface residues, our network analysis suggests the presence of interaction pathways to connect them. The P36L mutation thus indirectly affects the interface residues by changing the compactness of an interaction network.

**Figure 4:**
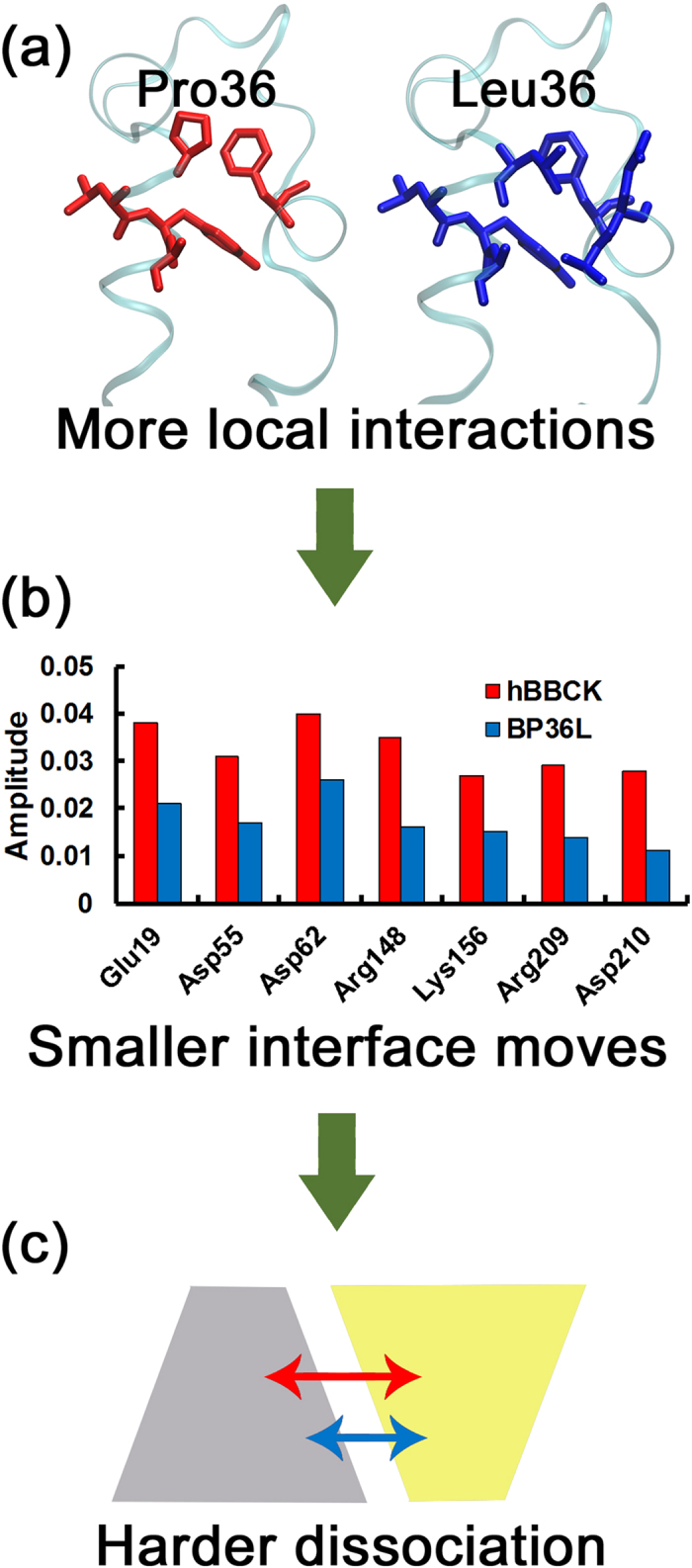
Molecular model to explain the isoenzyme-specific thermostability of human cytosolic CKs. When Pro36 (red) is mutated to Leu36 (blue), the more local interaction connections formed around residue 36 **(a)** can help suppress the motion of interface residues **(b)** through an interaction network, which finally inhibits the dimer dissociation **(c)**.

Based on these findings, we finally proposed a molecular model to explain the mechanism for the isoenzyme-specific thermostability of human cytosolic CKs (Fig. 4). The difference in thermal stabilities of the two isoenzymes arises mainly from the change of tendency in dimer dissociation induced by the single amino acid substitution at position 36. In the presence of Pro36, the relatively weak communication between residue 36 and the interface ones allows the latter to move in a relatively more flexible manner, which thereby facilitates the dimer dissociation and impairs the thermal stability. The P36L mutation, however, suppresses the movements of interface residues by reinforcing the residue interaction network. Consequently, Leu36 can effectively enhance the thermal stability of human cytosolic CKs by indirectly stabilizing the dimer interface. This model may shed light on the relationship between structural variation and protein stability as well as the evolution of the isoenzymes in adaptation to the physiological environment at the structural level.

### Thermal inactivation is partially caused by conformational changes other than dimer dissociation

The cMD simulations performed in our preceding analyses are incapable of monitoring the large-scale conformational change of proteins due to the limited simulation time. In the thermal inactivation experiment, however, the proteins are expected to greatly deviate from the native conformations by absorbing heat. To simulate such large-scale conformational changes, we performed accelerated MD (aMD) simulations^35^,^36^, a popular enhanced sampling technique, on the last snapshots of pre-equilibrations of cMD simulations for the four proteins including hBBCK, hMMCK, BP36L and ML36P (Table S3). All proteins undergo drastic conformational changes during the 200 ns aMD simulations. As shown in Fig. S10a, root-mean-square deviation (RMSD) of α-carbon atoms keeps rising in the aMD simulation of hBBCK to >12 Å, while the value remains relatively stable at ~5–6 Å for hMMCK. Meanwhile, the RMSD curves of BP36L and ML36P are between the two WT proteins. This order in the degree of structural deviation is consistent with the thermal inactivation experiment, because proteins experiencing larger conformational changes in enhanced sampling simulations are more prone to become denatured and inactivated upon heating. Further analysis shows that the large conformational change in hBBCK comes more from the inter-chain movement than from the intra-chain one, as indicated by RMSD and root-mean-square fluctuation (RMSF) curves for the single subunits (chain A and B) (Fig. S10c–f). However, the drastically changed conformation (called misfolded in the following discussion) observed in hBBCK does not reflect the dimer dissociation, since the distance between the centers of subunits drops greatly (Fig. S10b).

To identify the new conformation sampled in the aMD simulation of hBBCK, all structural snapshots were clustered according to pairwise RMSD values. The optimal cutoff value was chosen to allow the top 8 clusters to contain >90% of the structural snapshots (Table S11), and the structures closest to cluster centroids were taken as representatives of the clusters. Consistent with the above RMSD and domain distance calculations, the 3rd cluster in the hBBCK trajectory exhibits the largest conformational change with RMSD of 11.6 Å on average, while none of the other three proteins exceed 8 Å (Fig. S11a). Consequently, the representative structure of the 3rd cluster in hBBCK was taken as the major misfolded conformation after the large-scale structural change. As expected, the two subunits in this conformation rotate towards each other to form a collapsed structure (Fig. S12a), which explains the reduction in the distance between the subunit centers during the aMD simulation of hBBCK (Fig. S10b). In the hBBCK native structure, the opening and closing of the substrate binding site are controlled by a pair of flexible loops from the opposing subunits^16^. However, the representative misfolded structure of hBBCK completely blocks the substrate entrance in both chains through rotation of subunits, even when the pair of flexible loops adopts the open conformation (Fig. S13). As shown by the curves of radii calculated by HOLE2^37^,^38^, the narrowest point in the substrate entering pathway is less than 2.3 Å in chain A and is nearly zero in chain B (Fig. S14), thereby completely inhibiting the binding of substrates.

Therefore, apart from the effect of dimer dissociation, the thermal inactivation of hBBCK may partially arise from the formation of a misfolded structure that disallows the substrate binding. This effect, however, makes weak contribution to the thermal inactivation of the other three proteins, considering their relatively well-preserved conformations in the aMD simulations (Fig. S12).

### Inferences to protein design

Manipulation on the thermal stability of enzymes is one of the basic topics in protein design and engineering. Conventionally, the thermal stability is optimized through iterative random residue mutations and *in vitro* screening^39^,^40^,^41^,^42^. Recently, numerous bioinformatics tools have been developed to facilitate the rational design on the protein stability using evolutionary information^43^. In this work, we adopted an experimental strategy of systematic domain swapping and site-directed mutagenesis to identify and to validate the key amino acid residues in isoenzymes that significantly differ in thermostability. Heppel *et al.* used a similar approach to identify the key residues responsible for the difference in activities between two homologous enzymes TT2 and PAP4^44^. This method directly utilizes evolutionary information that is implicitly involved in the sequences of a pair of isoenzymes, and therefore can substantially reduce time and expense. Particularly, isoenzymes widely exist in nature, and isoenzyme-specific properties may be general due to the differential adaption to distinct tissue-specific environments^45^,^46^. Moreover, such an approach can be applied in a more general manner. As long as two homologous proteins exhibit significant difference in some biophysical property, the above procedure can be initiated to identify the determining residues, and the obtained information can facilitate the rational protein design in return.

Once the key residues are identified, computer simulations and biophysical examinations can be adopted to uncover and to validate the molecular mechanism respectively, and the in-depth understanding on mechanism can further improve the accuracy of rational protein design. Particularly, the simulation technique has exhibited its strength in mechanistic studies on protein stability. In several previous simulation studies, the structures and energies of proteins with different thermostabilities were compared, and the collective motion mode analysis was engaged to study the interacting relationship between residues^34^,^47^,^48^. Noteworthy, in this work, our computational model, which combines the analyses of functional modes and interaction networks to investigate the relationship between structure and stability, was uniquely designed by us and has never been adopted by previous studies to our knowledge.

### Limitations

Although the tendency of dimer dissociation was numerically evaluated for all proteins and statistically significant difference was identified between the proteins carrying Pro and those carrying Leu at position 36 in both hBBCK and hMMCK systems, direct observation of dimer dissociation, which might exceed the timescale of cMD or aMD simulations performed in this work, was not achieved. Longer and more enhanced sampling simulations should be conducted in the future to visualize the molecular details of the dimer dissociation process. In addition, the low absolute PCC values in the FMA analysis suggest the contribution of non-linear correlation in the collective motion. Other correlation measurements such as mutual information^32^ will be tested in the future work to remedy this omission.

## Conclusion

In this work, we systematically investigated the difference in thermostabilities between two isoforms of human cytosolic CKs, hBBCK and hMMCK. By combining domain swapping and mutual point mutation, the residue 36 was identified as the main contributor for the large difference in semi-inactivation temperatures between the two isoenzymes. We subsequently conducted MD simulations to explore the molecular mechanism of this phenomenon. Using free energy calculations, we found the higher propensity of dimer dissociation in proteins carrying Pro than those carrying Leu at position 36 and identified seven key interface residues. Through the subsequent analyses on functional modes and interaction networks, we affirmed the presence of conserved interaction pathways between residue 36 and these interface residues, which can facilitate the remote stabilization on dimer association by the enhanced local interactions around residue 36 introduced by the P36L mutation. Both simulation results and the proposed mechanism were validated by biophysical examinations. In summary, this work combined *in silico* computer simulations and *in vitro* biophysical examinations, and successfully uncovered the isoenzyme-specific thermostability in human cytosolic CKs at the mechanistic level.

## Methods

### Construction of CK chimeras and site-directed mutagenesis

The cloning of *hBCK* and *hMCK* genes has been described elsewhere^7^. Ten chimeras were constructed with the swapping of the corresponding segments in hMMCK or hBBCK (Fig. 1a). Except for mutating residue 36 to the acidic Asp residue, site-directed mutagenesis was carried out mutually, i.e. the amino acids in hMMCK were mutated into their counterparts in hBBCK, and vice versa. All these mutants were verified by sequencing. The genes of chimeras were cloned into pET21b expression vector (Novagen, Germany). All enzymes were expressed in *Escherichia coli* BL21 [DE3]-pLysS (Stratagene, Germany) and purified as described previously^7^. The detailed information can be found in the Supplementary Information.

### Thermal inactivation

Thermal inactivation of the enzymes was carried out using the same procedure as described elsewhere^49^. In brief, thermal inactivation was performed by incubating 0.2 mg/ml enzyme in 5 mM Tris-HCl, pH 8.0 at a given temperatures for 10 min, and then the residual activities of the samples were measured at 25 °C according to the pH-colorimetry method^3^ in the phosphocreatine formation direction. The gel filtration assays of the thermal inactivated samples were performed on a Superdex 200 10/300 GL column (Pharmacia, America), using an ÄKTA purifier as described in our previous paper^7^. The detailed information can be found in the Supplementary Information.

### Model building

Crystal structures without substrates were used in this work for the hBBCK (PDB ID: 3DRE) and hMMCK (PDB ID: 1I0E) systems. The missing residues in chain B of hBBCK (residues 321–329) were directly generated from the complete chain A. The gap residues in both chains of hMMCK (residues 323–331) were modeled by Modeller^50^,^51^,^52^ using the counterpart in chain A of hBBCK as template. Therefore, both structural are called modified crystal structures (or native structures) in this work. The missing residues in N-termini of hMMCK were not built because of their great flexibility that may destabilize the structures during simulations. Site-directed mutagenesis of residue 36 was implemented by VMD 1.9.1^53^ based on the modified crystal structures. The four proteins were then placed in water boxes containing ~29150 water molecules and neutralized with 0.01 mol/L NaCl.

### Simulation parameters

Amber12SB force field^54^ and TIP3P water model^55^ were engaged to quantify the atomic interactions. Both cMD and aMD^35^,^36^ simulations were run using NAMD 2.9^56^ with periodic boundary conditions (PBC) applied. The temperature was held at 298 K while the pressure was controlled at 1 atm. The time step was set to 2 fs and the SETTLE algorithm^57^ was used to enable the rigid bonds connected to all hydrogen atoms. All four proteins followed a 3-step pre-equilibration of 32.9 ns, the last snapshots of which were chosen as the start structure for 100 ns cMD and 200 ns aMD productive simulations (Table S3) without constraints. Dual boost potentials^58^ were applied in aMD simulations according to previous works^59^,^60^. The detailed information can be found in the Supplementary Information.

### Analysis methods

Dimeric binding free energies were calculated using the MM/PBSA method implemented in AMBER12^61^. FMA based on partial least-squares algorithm^32^,^33^ was implemented using GROMACS 5.1-dev^62^ to find out the collective movements related with dimer dissociation. PCA was performed on α-carbon atoms only, using ProDy^63^. Network analysis was performed using NetworkX^64^ to identify the interaction pathways between residue 36 and key interface ones. Other parameter values for network construction were also tested. Clustering analysis was carried out using VMD 1.9.1^53^ according to pairwise RMSD values. The radii of substrate entering pathway to the active site were calculated by HOLE2^37^,^38^. The detailed information can be found in the Supplementary Information.

## Additional Information

**How to cite this article**: Liu, H. *et al.* A single residue substitution accounts for the significant difference in thermostability between two isoforms of human cytosolic creatine kinase. *Sci. Rep.* **6**, 21191; doi: 10.1038/srep21191 (2016).

## Supplementary Material

Supplementary Information

## Figures and Tables

**Table 1:** Binding free energies for the four proteins.

ID	ΔG (kcal/mol)	Frames used			p-value
		80 ns	90 ns	100 ns	
1	hBBCK	−179 ± 32	−181 ± 32	−181 ± 31	< 2.2e-16
2	BP36L	−198 ± 25	−200 ± 24	−202 ± 24	
3	hMMCK	−191 ± 20	−192 ± 20	−192 ± 20	< 2.2e-16
4	ML36P	−168 ± 30	−165 ± 30	−165 ± 29	

**Table 2:** Comparisons on motion amplitudes and directions of residue 36 and seven key interface residues in the PC2 mode of hBBCK and that of BP36L.

		Amplitude		Correlation in directions
Key residue		hBBCK	BP36L	
Chain A	Pro/Leu36	0.035	0.020	0.96
	Glu19	0.038	0.021	0.99
	Asp55	0.031	0.017	0.97
	Asp62	0.040	0.026	0.96
	Arg148	0.035	0.016	0.98
	Lys156	0.027	0.015	0.97
	Arg209	0.029	0.014	0.97
	Asp210	0.028	0.011	0.96
Chain B	Pro/Leu36	0.009	0.009	0.88
	Glu19	0.028	0.014	0.98
	Asp55	0.025	0.017	0.95
	Asp62	0.031	0.015	0.90
	Arg148	0.033	0.019	0.97
	Lys156	0.018	0.020	0.99
	Arg209	0.047	0.012	1.00
	Asp210	0.044	0.020	0.98

The correlation between two corresponding residues is defined as the cosine value of the angle between their motion directions, which ranges from −1 to 1.

**Table 3:** Comparison of network properties.

Property	hBBCK		BP36L	
	Chain A	Chain B	Chain A	Chain B
Number of nodes	367	363	371	367
Number of edges	953	872	996	953
Degree of residue 36	4	2	6	6
Average node degree	5.193	4.804	5.369	5.193
Average clustering coefficient	0.212	0.192	0.220	0.223
