# Supplementary material for: A single residue substitution accounts for the significant difference in thermostability between two isoforms of human cytosolic creatine kinase
Source: Sci Rep. 2016 Feb 16;6:21191. doi: 10.1038/srep21191 (PMC4754747; doi:10.1038/srep21191)
Supplement: Supplementary Information [file srep21191-s1.pdf]

Supplementary Information

A single residue substitution accounts for the significant difference in thermostability between two isoforms of human cytosolic creatine kinase

Huihui Liu[1,#], Yan-Song Gao[2,3,#], Xiang-Jun Chen[2], Zhe Chen[2,3], Hai-Meng Zhou[3], Yong-Bin Yan[2,*] and Haipeng Gong[1,*]

[1]MOE Key Laboratory of Bioinformatics, School of Life Sciences, Tsinghua University, Beijing 100084, China

[2]State Key Laboratory of Membrane Biology, School of Life Sciences, Tsinghua University, Beijing 100084, China

[3]Zhejiang Provincial Key Laboratory of Applied Enzymology, Yangtze Delta Region Institute of Tsinghua University, Jiaxing 314006, China

[#]These authors contributed equally to this work.

[*]To whom correspondence should be addressed:

Haipeng Gong, School of Life Sciences, Tsinghua University, Beijing 100084, China, Tel: +86-10-6279-4753; Email: hgong@tsinghua.edu.cn

Yong-bin Yan, School of Life Sciences, Tsinghua University, Beijing 100084, China, Tel: +86-10-6278-3477; Email: ybyan@tsinghua.edu.cn

Supplementary methods

Chemicals

Sodium dodecylsulfate (SDS), isopropyl-1-thio-β-D-galactopyranoside (IPTG), Tris, creatine, DTT and ATP were purchased from Sigma. The restriction enzymes were obtained from New England Biolabs or TaKaRa. All other chemicals were local products of analytical grade.

Construction of CK chimeras and site-directed mutagenesis

The cloning of *hBCK* and *hMCK* genes has been described elsewhere[1]. Ten chimeras (MnBc, BnMc, $M_{1\text{-}53}B$, $B_{1\text{-}53}M$, $M_{1\text{-}53}B_{54\text{-}116}M$, $B_{1\text{-}53}M_{54\text{-}116}B$, $M_{1\text{-}26}B$, $B_{1\text{-}26}M$, $M_{1\text{-}26}B_{27\text{-}53}M$ and $B_{1\text{-}26}M_{27\text{-}53}B$) were constructed with the swapping of the corresponding segments in hMMCK or hBBCK (Fig. 1a). Except for mutating residue 36 to the acidic Asp residue, site-directed mutagenesis was carried out mutually, i.e. the amino acids in hMMCK were mutated into their counterparts in hBBCK, and vice versa. The hBBCK-derived mutants included BP36D, BP36L, BA40K, BE41K, BA44D, BE46S and BL53V, while the hMMCK-derived mutants involved ML36D, ML36P, MK40A, MK41E, MD44A, ME46S and MV53L. All these mutants were verified by sequencing. The genes of chimeras were cloned into pET21b expression vector (Novagen, Germany). The primers used for the constructs (Tables S12 and S13) can be supplied upon request.

Protein expression and purification

All enzymes were expressed in *Escherichia coli* BL21 [DE3]-pLysS (Stratagene, Germany) and purified as described previously[1]. In brief, the overexpression of the recombinant proteins was induced by 0.4 mM IPTG. After 24 h incubation at 16 °C, the cells were harvested and lysed by lysozyme. The recombinant proteins were separated from the supernatant of the cell lysate using a DEAE Sepharose Fast Flow anion-exchange column. The final products were collected from a Sephacryl S300 HR column equipped on an AKTA purifier. The purities of native as well as all recombinant proteins exceeded 95%, as evaluated by SDS-PAGE and SEC analysis. The protein concentration was determined according to the Bradford method by using bovine serum albumin as a standard[2].

Activity assay

The activity of CK was measured at 25°C according to the pH-colorimetry method[3] in the phosphocreatine formation direction by monitoring the absorbance changes at 597 nm on an Ultraspec-4300 Pro spectrophotometer (Pharmacia, America). All the activity experiments were repeated for at least three times.

Thermal inactivation

Thermal inactivation of the enzymes was carried out using the same procedure as described elsewhere[4]. In brief, thermal inactivation was performed by incubating 0.2 mg/ml enzyme in 5 mM Tris-HCl, pH 8.0 at given temperatures for 10 min, and then the residual activities of the samples were measured. The gel filtration assays of the

thermal inactivated samples were performed on a Superdex 200 10/300 GL column (Pharmacia, America), using an ÄKTA purifier as described in our previous paper[1].

Model building

Crystal structures without substrates were used in this work for the hBBCK (PDB ID: 3DRE) and hMMCK (PDB ID: 1I0E) systems. The missing residues in chain B of hBBCK (residues 321-329) were directly generated from the complete chain A. The gap residues in both chains of hMMCK (residues 323-331) were modeled by Modeller[5-7] using the counterpart in chain A of hBBCK as template. Therefore, both structural are called modified crystal structures (or native structures) in this work. The missing residues in N-termini of hMMCK were not built because of their great flexibility that may destabilize the structures during simulations. Site-directed mutagenesis of residue 36 was implemented by VMD 1.9.1[8] based on the modified crystal structures. The four proteins were then placed in water boxes containing ~29150 water molecules and neutralized with 0.01 mol/L NaCl.

Simulation parameters

Amber12SB force field[9] and TIP3P water model[10] were engaged to quantify the atomic interactions. Both cMD and aMD[11,12] simulations were run using NAMD 2.9[13] with periodic boundary conditions (PBC) applied. The temperature was held at 298 K using the Langevin thermostat[14], while the pressure was controlled at 1 atm by Berendsen pressure bath coupling method[15]. Particle mesh Ewald (PME) method[16]

was used to calculate electrostatics. The van der Waals interactions were truncated at 9.0 Å with a long-range correction. The time step was set to 2 fs for both cMD and aMD simulations and the SETTLE algorithm[17] was used to enable the rigid bonds connected to all hydrogen atoms.

All four proteins followed a 3-step pre-equilibration. Firstly, all heavy atoms of proteins except the modelled loops were constrained with a force constant of 10 kcal/mol/Å^2 for 0.5 ns to allow the reasonable redistribution of water and ions. Secondly, backbone atoms except the modelled loops were constrained with the restraint constant gradually relaxed from 10 to 0 kcal/mol/Å^2 in 2.4 ns. Finally, 30ns simulation without constraints was carried out. The last snapshot of pre-equilibration was chosen as the start structure for cMD and aMD productive simulations of each protein. No constraint was imposed on proteins during productive simulations, which lasted 100 and 200 ns for cMD and aMD simulations respectively (Table S3). Structures were saved every 20 ps and diluted 5 times for further analysis.

Dual boost potentials[18] were applied independently to the dihedral and total energies in aMD simulations according to previous works[19,20], in the following manner,

$$\begin{aligned} E_d &= \langle V_d \rangle + 4 \cdot n_{res} \\ \alpha_d &= 0.8 \cdot n_{res} \\ E_t &= \langle V_t \rangle + 0.2 \cdot n_{atom} \\ \alpha_t &= 0.2 \cdot n_{atom} \end{aligned}, \quad (1)$$

where E_d and E_t are the threshold energies for dihedral and total potentials, α_d and α_t are the corresponding acceleration factors, and n_{res} and n_{atom} represent the numbers of protein residues and the total system atoms respectively. The average dihedral and

total potential energies, $<V_d>$ and $<V_t>$, were calculated from the last 10 ns of the cMD pre-equilibrations.

Binding free energy calculation

Dimeric binding free energies were calculated using the MM/PBSA method implemented in AMBER12[21]. 1000 frames from the diluted 100 ns cMD simulations were taken as input structures. Entropies were omitted considering the similar structures of all proteins. PB calculations for polar solvation free energies were performed using internal PBSA solver[22]. Dielectric constants for the interior and exterior of the molecule were set to 1 and 80 respectively. Nonpolar solvation free energies were evaluated by the SASA and a surface-integration method for the repulsive and attractive terms respectively. Atoms radii for the PB calculation were taken from the values optimized by Tan and Luo[23]. Solvent probe radius was 1.4 Å. Binding free energies were further decomposed to each residue with 1-4 terms added to internal potential terms.

Movement mode analysis (FMA and PCA)

FMA based on partial least-squares algorithm[24,25] was implemented using GROMACS 5.1-dev[26] to find out the collective movements related with dimer dissociation. The biological function was defined as the total SASA of all interface residues in one subunit, which was the half of SASA difference between the two separated chains and the dimeric structure. Atom radii from the AMBER12 topology

files were used to calculate SASA by VMD 1.9.1[8]. The starting structures for productive simulations were used as references and only α-carbon atoms were fitted and analyzed. The ensemble-weighted mode was used to generate the functional mode.

PCA was performed on α-carbon atoms only, using ProDy[27]. The top 20 modes were retained for further analysis.

Network analysis

Network analysis was performed to identify the interaction pathways between residue 36 and key interface ones. To construct the network, each residue was treated as a node and two nodes were connected only when the following two criteria were satisfied simultaneously: 1) the moving directions of the two residues in the PC2 mode have a scalar angle < 45°; 2) the two residues make physical contact, which requires the presence of at least one pair of heavy atoms from two candidate residues approximating to a distance of < 4.5 Å in at least 75% of the frames in the overall simulation trajectory[28,29]. The connections between adjacent residues were removed[28,30]. The generated undirected unweighted networks were analyzed using NetworkX[31]. The node degree describes the number of edges connected to one node. The clustering coefficient of each node refers to the fraction of triangles that indeed exist in the network over all theoretically possible triangles in its neighborhood. The above two properties are averaged over all nodes to estimate the average degree and average clustering coefficient of the network respectively[32]. 10 repeated calculations

were performed in a bootstrap manner to estimate the statistical significance: 20% frames were randomly removed from the productive trajectory for each time.

Considering the dependence of the constructed network upon the criterion parameters, other parameter values were also tested. The scalar angle between moving directions of two residues was set to 40°, 45°, 50° and 55°, the distance cutoff between two heavy atoms was tested at 4.0, 4.5, 5.0 and 5.5 Å, and the ratio cutoff (percentage in the trajectory) was evaluated for 65%, 70%, 75% and 80%. The results are shown in Tables S14-16. The difference between hBBCK and BP36L is robust and significant for most of the tested combinations of parameter values.

Clustering analysis

Clustering analysis was carried out using VMD 1.9.1[8] according to pairwise RMSD values. The optimal cutoff value was chosen to allow the top 8 clusters to contain >90% of the structural snapshots[33], which is 3.0 Å in BP36L and 2.8 Å in the other proteins (Table S11). The structures closest to cluster centroids were taken as representatives of the clusters.

The radii of substrate entering pathway to the active site were calculated by HOLE2[34,35] and the graphs were prepared using VMD 1.9.1[8]. Default simple atom radii were used[36].

Supplementary figures

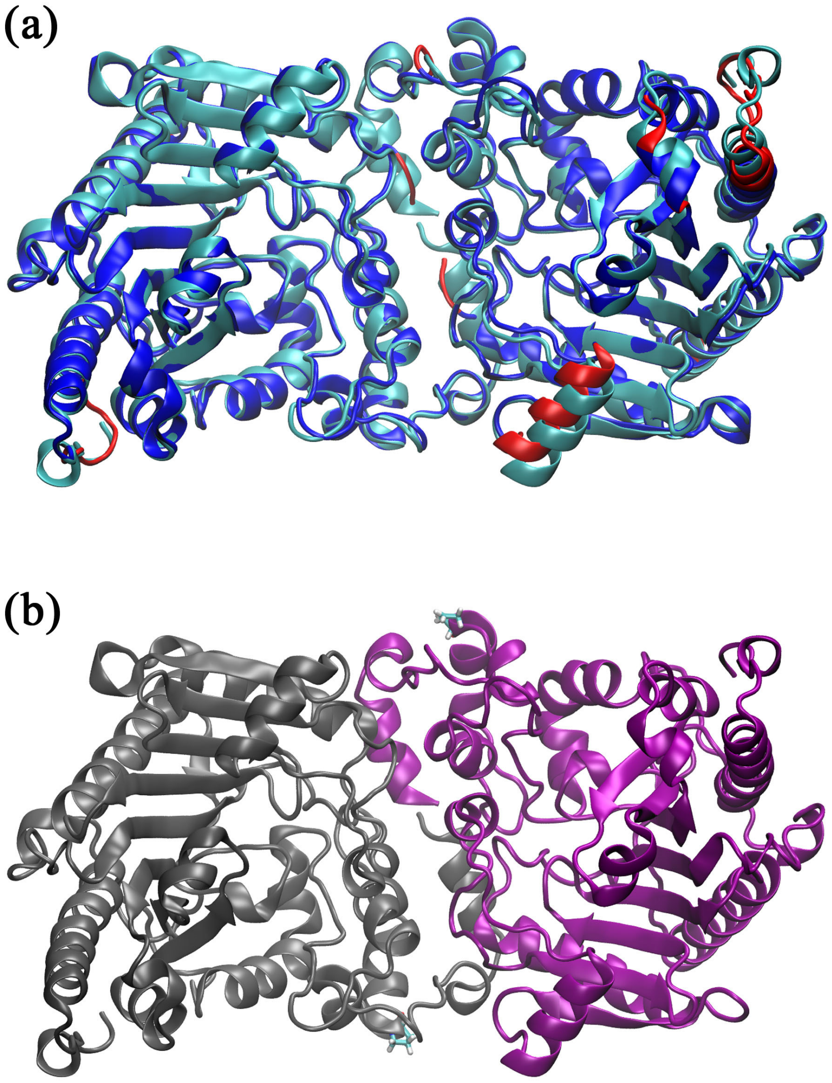

Fig. S1. (a) Superposition of the dimeric model structures of hBBCK (cyan) and hMMCK (blue). The structural models were generated from the crystal structure, as described in *Methods*. The regions showing major differences (root mean square deviation (RMSD) > 2 Å) between the two isoenzymes are highlighted in red in the hMMCK structure. **(b)** The structure of hBBCK with chain A colored in gray and

chain B colored in purple. The residue 36 is shown in the Licorice representation in both subunits. All structural figures were made using VMD 1.9.1[8].

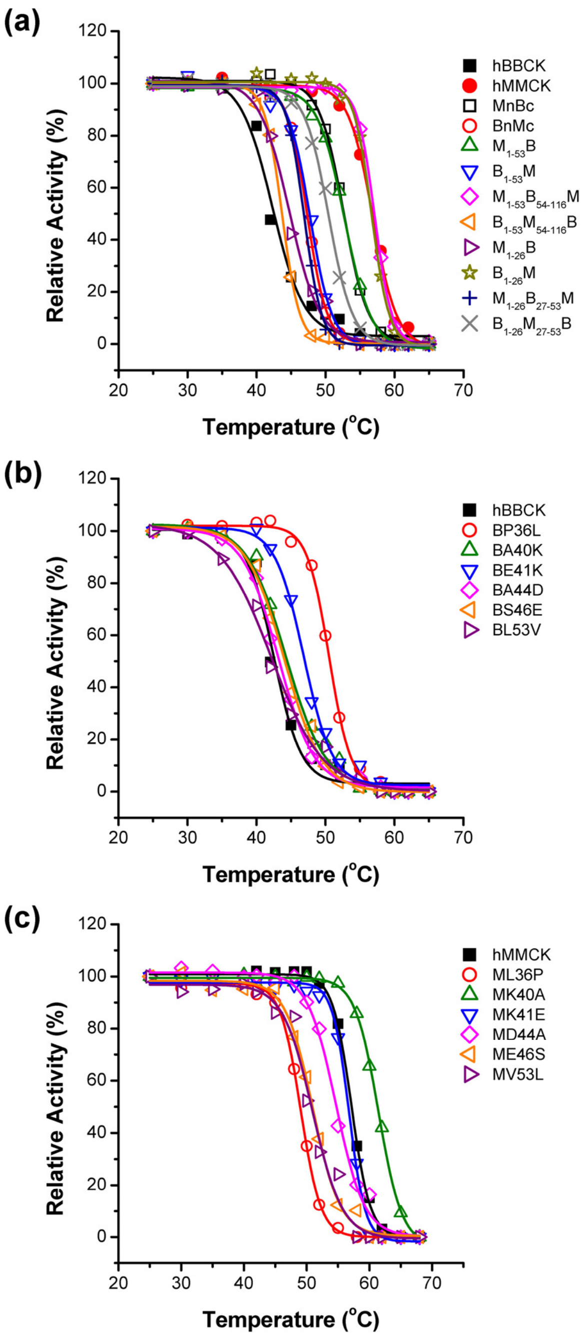

Fig. S2. The thermal inactivation curves for **(a)** WT CKs and chimeras, **(b)** hMMCK and its mutants and **(c)** hBBCK and its mutants.

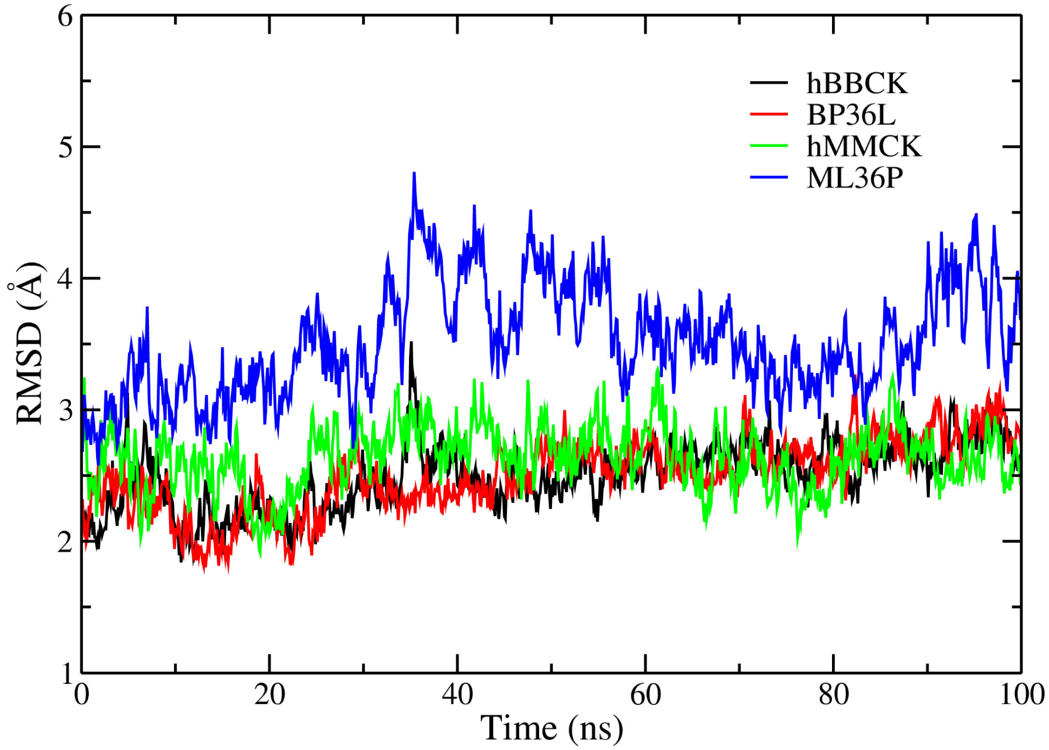

Fig. S3. Time series of RMSD for α-carbon atoms in the cMD productive simulations of the four proteins: hBBCK (black), BP36L (red), hMMCK (green) and ML36P (blue).

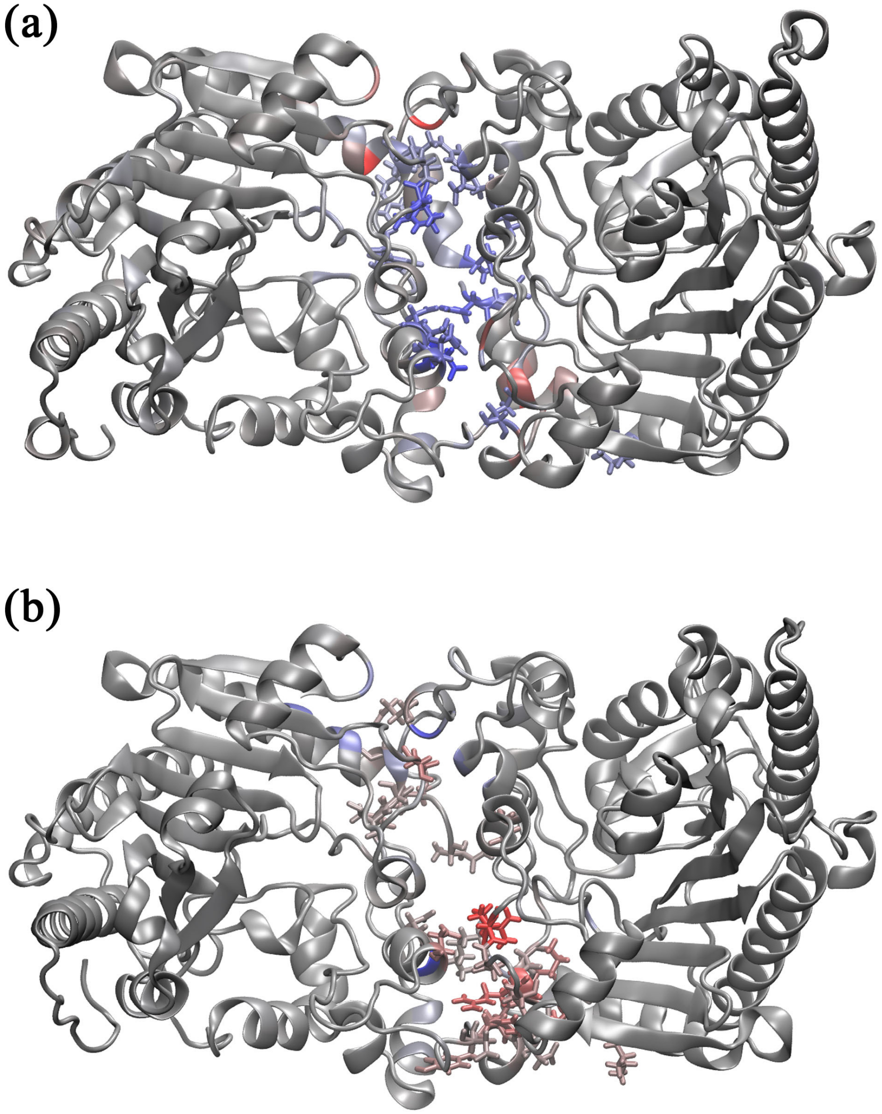

Fig. S4. Residues that make significantly different contributions to the binding free energies between the mutants and WTs of hBBCK and hMMCK. **(a)** Residues that selectively stabilizes BP36L over hBBCK. **(b)** Residues that selectively destabilizes ML36P over hMMCK. The residues are shown in the Licorice representation, in the color to reflect the degree of difference in the binding free energies between mutant and WT isoenzymes. In specific, blue means increase of residue contribution in

mutants as compared to WT, while red indicates the reverse. The overall proteins are shown in the Cartoon representation and colored in gray.

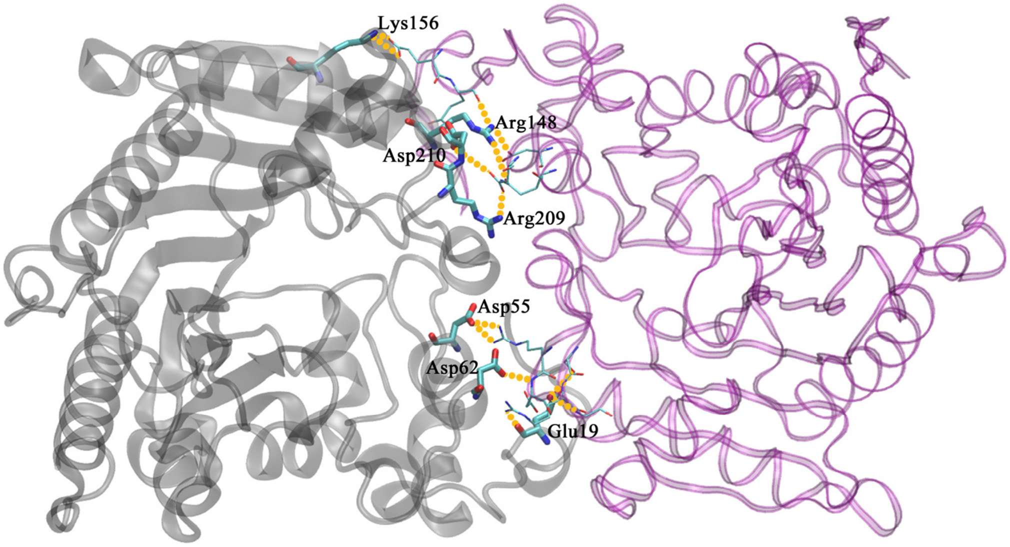

Fig. S5. The seven key interface residues (thick Licorice) and their interacting partners (thin Licorice) in the other subunit. The dotted yellow lines denote hydrogen bonds. The two subunits are shown as gray cartoon and pink ribbon respectively. The key interface residues are shown in only one subunit for clarity. The structure was taken from cMD trajectory of hMMCK.

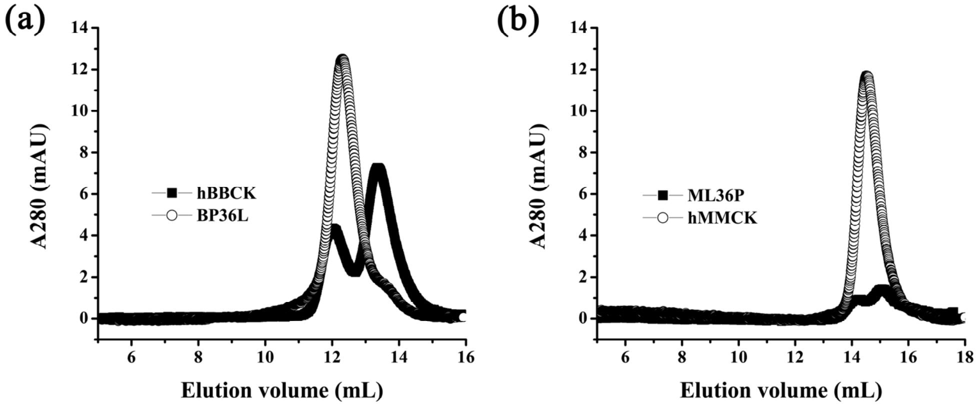

Fig. S6. The dimer dissociation detected during heat inactivation. **(a)** Comparison on the components of hBBCK (filled square) and BP36L (open circle) heated at 45 °C. **(b)** Comparison on the components of hMMCK (open circle) and ML36P (filled square) heated at 52 °C. The presence of protein components was detected using light absorbance at 280 nm.

(a) (b)

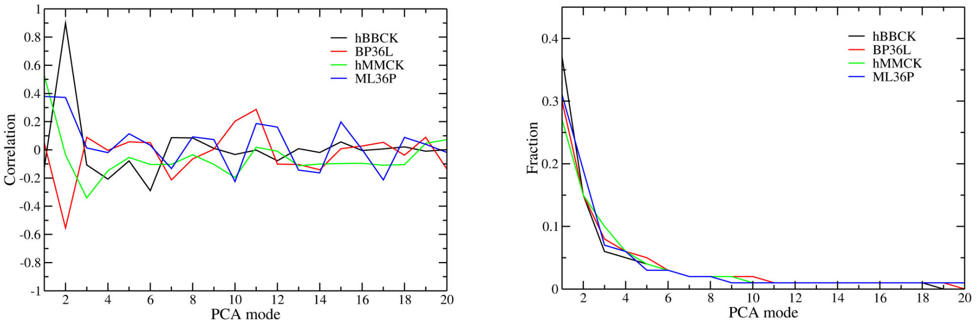

Fig. S7. Comparison on the functional modes of four proteins using FMA analysis. **(a)** Correlation coefficients between the functional mode of hBBCK and top 20 PCs of four proteins. **(b)** Fraction of the top 20 PCs in the four systems. The hBBCK, BP36L, hMMCK and ML36P are colored in black, red, green and blue, respectively.

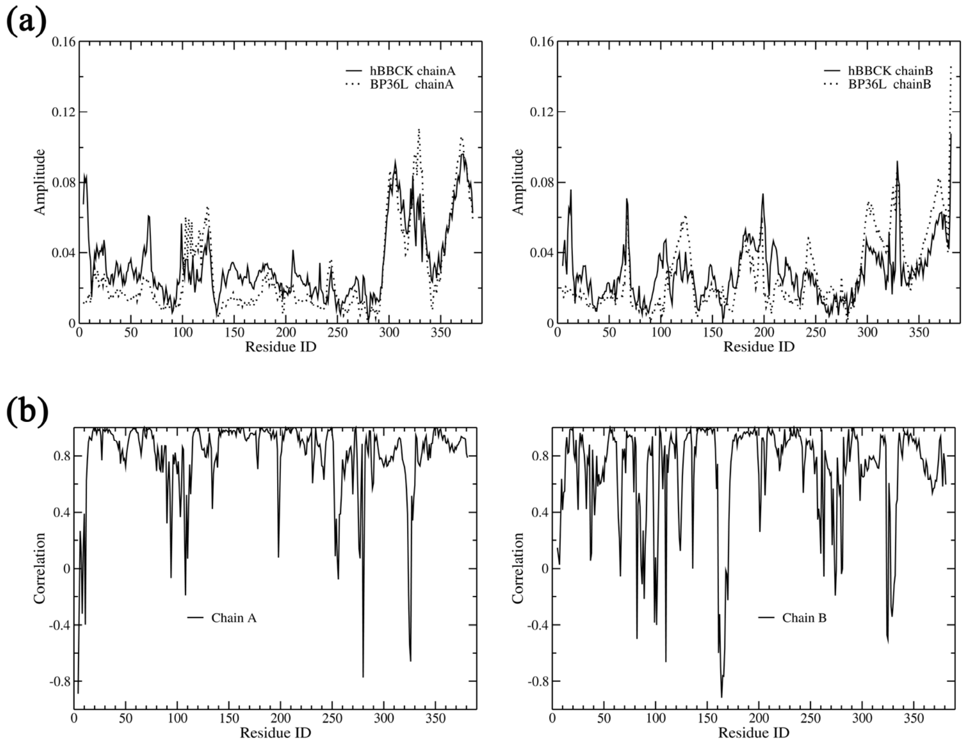

Fig. S8. Comparison on the motion of residues in the PC2 mode of hBBCK and that of BP36L. **(a)** The amplitudes of residue motion in hBBCK (solid) and BP36L (dotted). **(b)** The correlation coefficients between the moving directions of residues in hBBCK and BP36L. The results for chain A and chain B are shown in the left and right panels respectively.

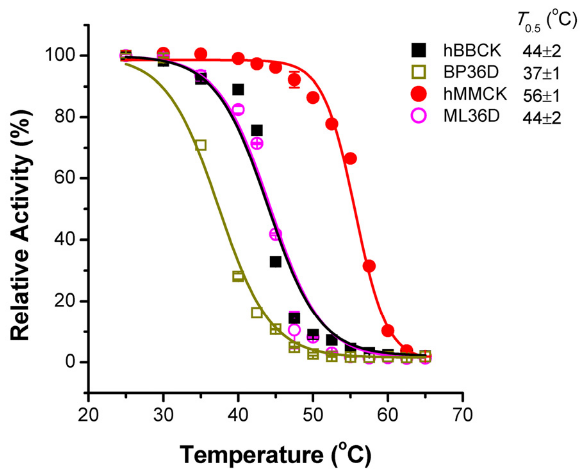

Fig. S9. The thermal inactivation curves for the mutants where the residue 36 was mutated to Asp in both hBBCK (square) and hMMCK (circle) systems. The error bar at each data point was derived from three independent thermal inactivation experiments conducted at the same temperature.

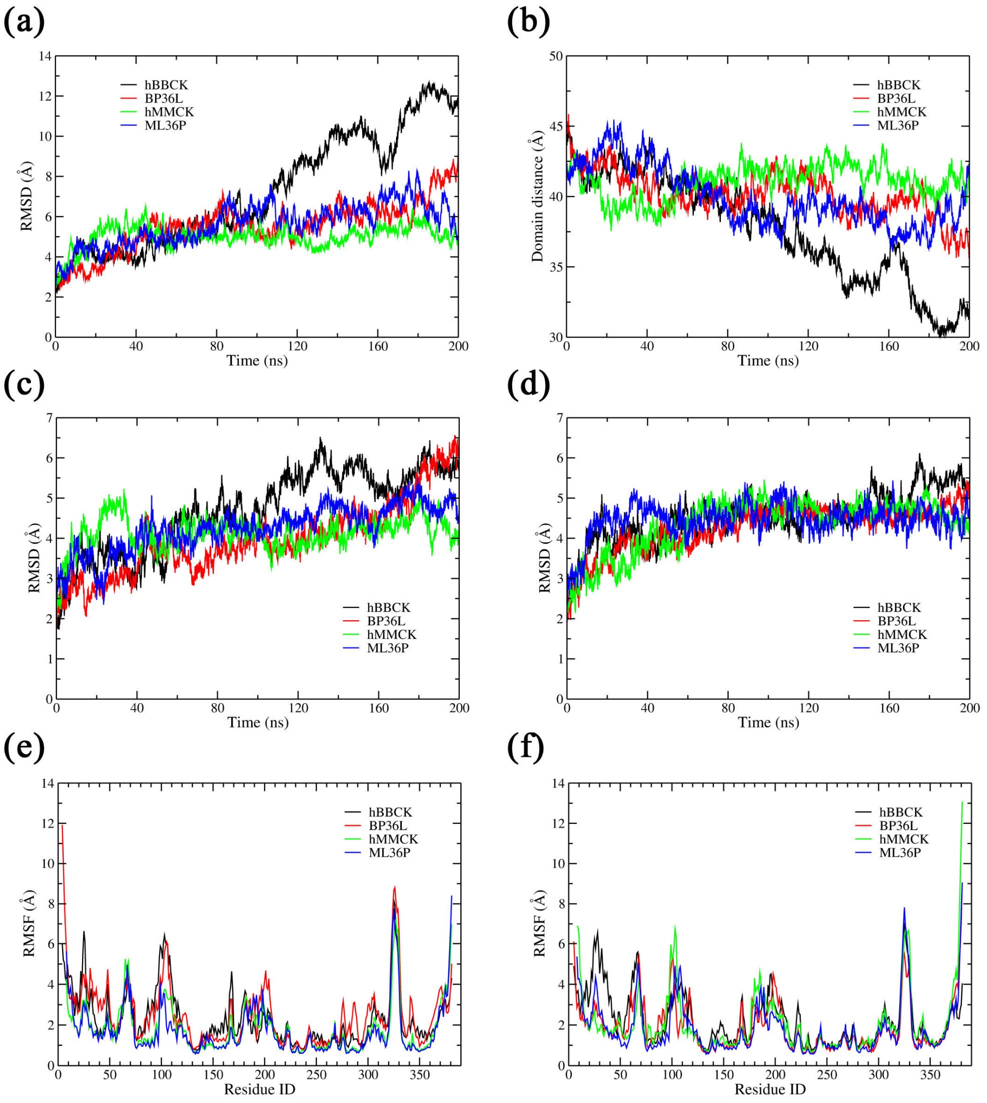

Fig. S10. Structural changes of the four proteins during the 200 ns aMD simulations. **(a)** The time series of RMSD of α-carbon atoms. **(b)** The time series of the distance between the centers of heavy atoms in chain A and chain B. **(c, d)** The time series of RMSD of α-carbon atoms in chain A (**c**) and chain B (**d**), respectively. **(e, f)** RMSF of α-carbon atoms in chain A (**e**) and chain B (**f**), respectively. The hBBCK, BP36L, hMMCK and ML36P are colored in black, red, green and blue, respectively.

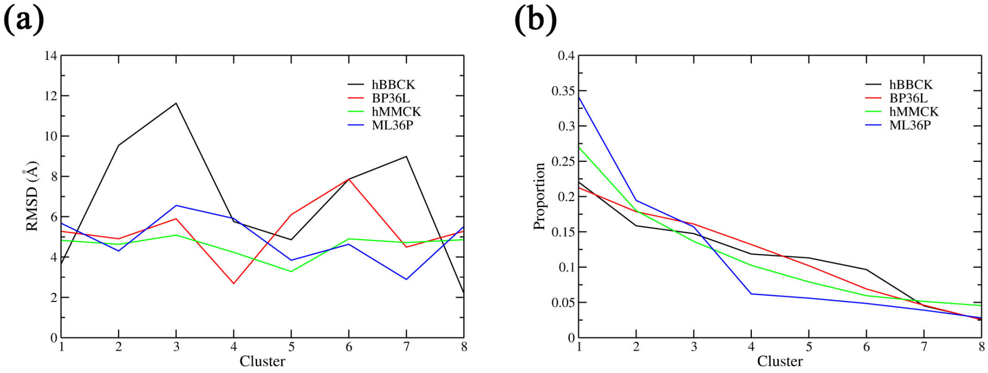

Fig. S11. (a) RMSD of α-carbon atoms for the average structure of each cluster in the aMD trajectories compared to the initial (native) structure. **(b)** Proportion of frames in each cluster. The hBBCK, BP36L, hMMCK and ML36P are colored in black, red, green and blue, respectively.

(a) (b)

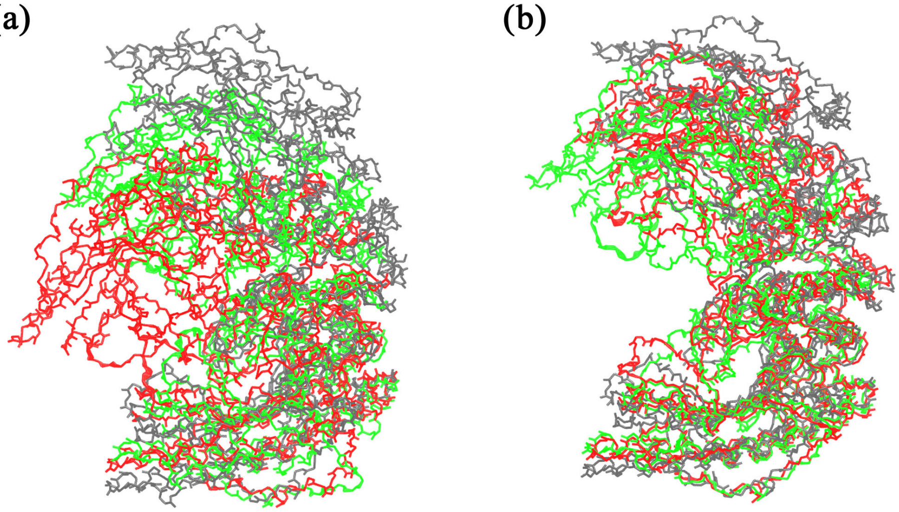

Fig. S12. Superposition of aMD structures against the modified crystal structures. **(a)** Structure comparison in the hBBCK system. The aMD cluster centroids deviating most from the modified crystal structure (gray) are shown in red and green for hBBCK and BP36L respectively. **(b)** Structure comparison in the hMMCK system. The aMD cluster centroids deviating the most from the modified crystal structure (gray) are shown in red and green for hMMCK and ML36P respectively.

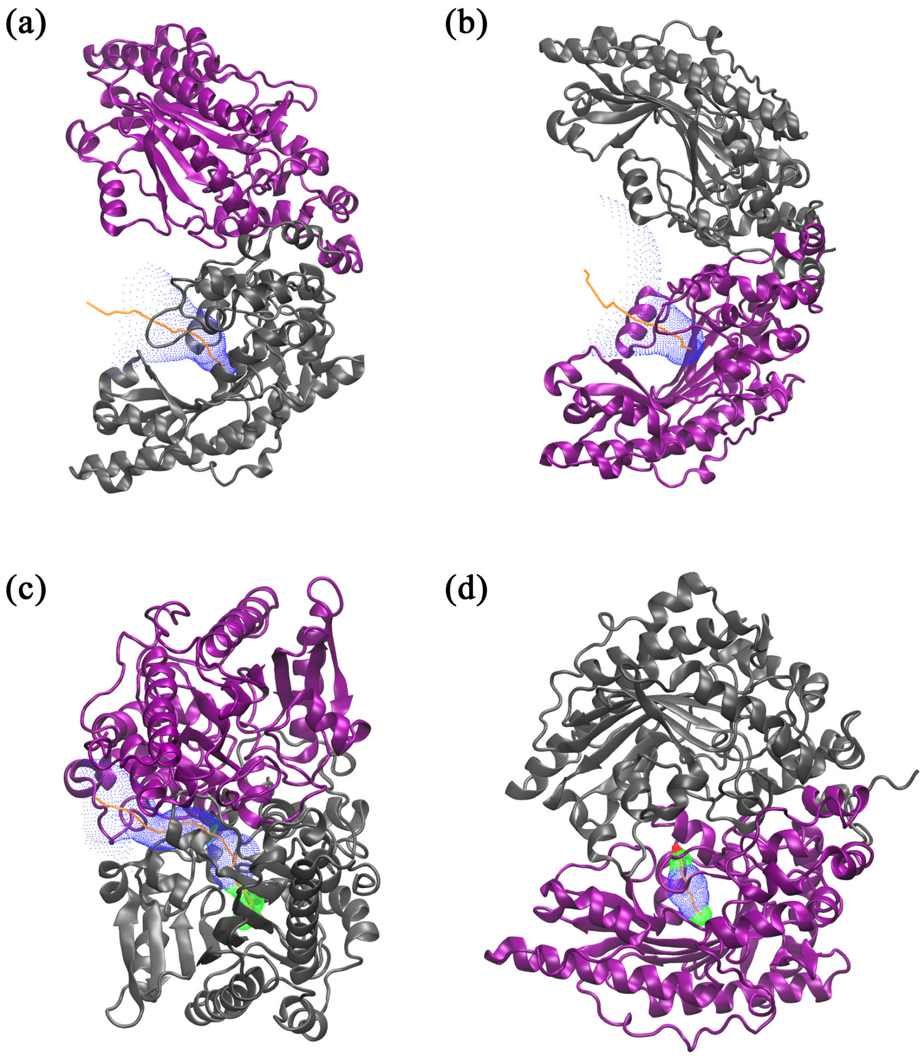

Fig. S13. Accessibilities of the substrate binding sites in the modified crystal structure **(a, b)** and the major misfolded structure **(c, d)** of hBBCK. The substrate entering pathways were evaluated for chain A (**a**, **c**, gray cartoon) and chain B (**b**, **d**, purple cartoon) respectively. The pore is shown in point diagram, colored red for radii < 1.15 Å, blue for radii > 2.3 Å, and green for radii between 1.15 and 2.3 Å. Orange lines represent the centers of the pore profiles.

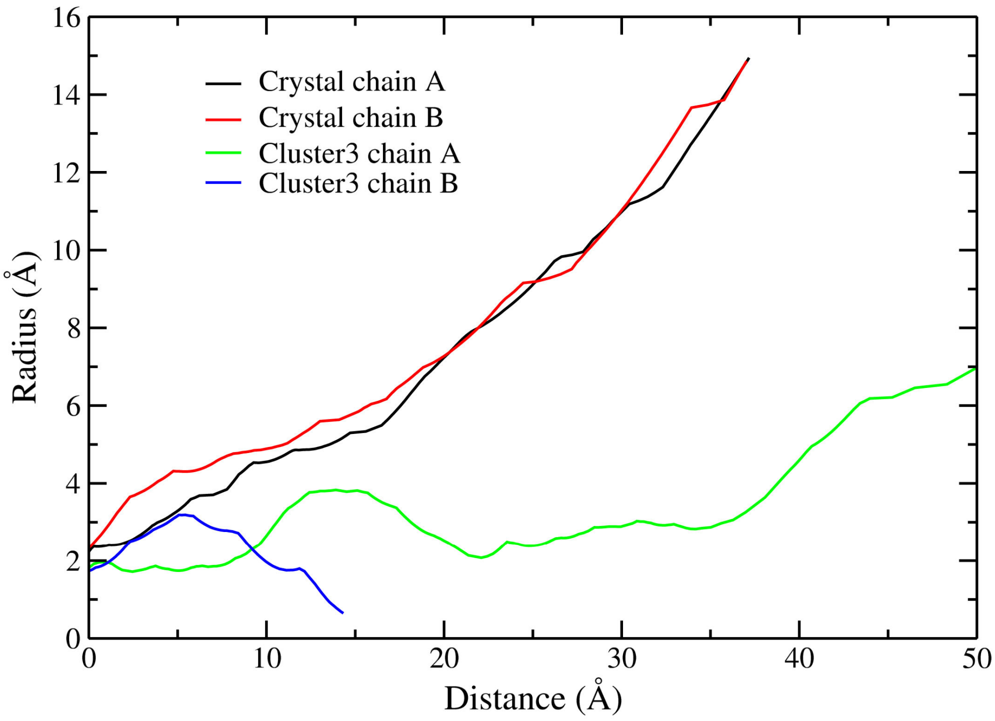

Fig. S14. Radii along the substrate entering pathway to the active site in hBBCK. The active site was set as the destination of the pathway (zero in the horizontal axis), and the positions of all other places were evaluated by their distances to this reference. The curves for chain A and chain B in the modified crystal structure are colored in black and red respectively, while those for chain A and chain B in the representative structure of the third cluster (or major misfolded structure) in the aMD trajectory of hBBCK are colored in green and blue respectively.

Supplementary tables

Table S1. Semi-inactivation temperatures and relative activities of hBBCK, hMMCK and their chimeras

Enzyme	Semi-inactivation Temperature (°C)	Relative activity (%)
hBBCK	42.3±0.4	100
hMMCK	56.8±0.2	52±2
MnBc	52.6±0.1	89±2
BnMc	47.3±0.1	66±1
$M_{1\text{-}53}B$	52.7±0.1	73±2
$B_{1\text{-}53}M$	47.7±0.2	68±1
$M_{1\text{-}53}B_{54\text{-}116}M$	57.1±0.1	63±1
$B_{1\text{-}53}M_{54\text{-}116}B$	43.7±0.1	67±2
$M_{1\text{-}26}B$	44.8±0.3	96±2
$B_{1\text{-}26}M$	56.7±0.1	72±1
$M_{1\text{-}26}B_{27\text{-}53}M$	46.9±0.1	62±1
$B_{1\text{-}26}M_{27\text{-}53}B$	50.4±0.1	94±1

All activities were normalized to that of hBBCK at 25°C, which was taken as 100%. Data for hBBCK and hMMCK were taken from our previous work[1].

Table S2. Semi-inactivation temperatures and relative activities of the site-directed mutants of hBBCK and hMMCK

Mutant name	Semi-inactivation Temperature (°C)	Relative activity (%)
Group I		
BP36L	50.5±0.1	95±1
BA40K	44.3±0.4	111±1
BE41K	46.9±0.2	100±1
BA44D	43.2±0.2	114±1
BS46E	44.0±0.2	101±1
BL53V	42.2±0.4	96±1
Group II		
ML36P	49.1±0.1	84±1
MK40A	61.5±0.1	109±2
MK41E	56.7±0.2	141±2
MD44A	54.7±0.3	83±1
ME46S	51.0±0.2	86±1
MV53L	51.0±0.4	44±1

Relative activity was expressed as the percentage to hBBCK and hMMCK at 25°C in Group I and Group II, respectively.

Table S3. The information of MD simulations

ID	System	Pre-equilibration (ns)	cMD simulation (ns)	aMD simulation (ns)
1	hBBCK	30	100	200
2	BP36L	30	100	200
3	hMMCK	30	100	200
4	ML36P	30	100	200

Table S4. Difference in the binding free energies between the mutants and the WTs of human cytosolic CKs

Residue in hBBCK	ΔΔG(BP36L-hBBCK) (kcal/mol)	Residue in hMMCK	ΔΔG(ML36P-hMMCK) (kcal/mol)
A Asp 54	-5.47	**B Arg 209**	18.73
**A Arg 209**	-3.75	**B Arg 148**	7.81
A Asn 5	-3.64	B Arg 152	6.21
B Asn 5	-3.14	A Lys 11	5.56
**A Asp 55**	-2.62	**A Asp 210**	4.87
**A Asp 62**	-2.45	**A Glu 19**	4.63
A Gln 58	-2.44	**A Asp 62**	3.93
A Ser 6	-2.42	A Tyr 20	3.07
**A Glu 19**	-2.03	**B Asp 55**	2.92
**B Asp 55**	-2.00	B Asp 213	2.81
**B Asp 62**	-1.70	B Lys 11	2.51
A Ser 49	-1.57	**B Lys 156**	2.41
A His 145	-1.57	**B Asp 210**	2.28
B Asp 54	-1.57	B Glu 18	1.93
**B Lys 156**	-1.39	A Glu 18	1.69
**A Arg 148**	-1.32	A Gly 149	1.65
**A Asp 210**	-1.14	A Ser 147	1.61
B Arg 13	-1.11	B Ser 147	1.61
		**A Asp 55**	1.60
		A Asp 22	1.53
		A Tyr 14	1.51
		B Asn 8	1.44
		A Cys 146	1.40
		A Lys 9	1.10

Each residue is denoted by its chain name, residue name and residue ID, separated by whitespace. A total of seven common key interface residues (bold) are identified from the hBBCK and hMMCK systems.

Table S5. Inter-chain interactions of the seven key interface residues

Key interface residue		hBBCK		BP36L		hMMCK		ML36P	
		Salt bridge	H-bond	Salt bridge	H-bond	Salt bridge	H-bond	Salt bridge	H-bond
Chain A	Glu19	—	Ser147, Arg148, Gly149	—	Ser147, Arg148, Gly149	—	Ser147, Arg148, Gly149	—	Ser147, Arg148, Gly149
	Asp55	Arg209	Arg209	Arg209	Arg209	Arg209	Arg209	Arg209	Asn8
	Asp62	Arg209	Arg209, Asp210	—	Asp210	—	Asp210	Arg209	Asp210
	Arg148	Asp54	Glu19, Gln58	Asp54	Glu19, Gln58	Asp54	Glu19, Asp54	Asp54	Glu19, Asp54
	Lys156	Glu17, Asp18	—	Asp18	—	Glu18	—	Glu18	Glu18
	Arg209	Asp54, Asp55	Asp55	Asp55	Asp55	Asp62	Asn8, Asp62, Ala204	Asp54, Asp55	Asp54, Asp55
	Asp210	—	Gln58, Asp62	—	Gln58, Asp62	—	Asp62	—	Gln58, Asp62
Chain B	Glu19	—	Ser147, Arg148, Gly149	—	Ser147, Arg148, Gly149	—	Ser147, Arg148, Gly149	—	Ser147, Arg148, Gly149
	Asp55	Arg209	Arg209	Arg209	Arg209	—	—	Lys9, Arg209	Arg209
	Asp62	—	Asp210	—	Asp210	Arg209	Arg209, Asp210	—	Asp210
	Arg148	Asp54	Glu19, Asp54	Asp54	Glu19	Asp54	Glu19, Asp54	Asp54	Glu19, Gln58
	Lys156	Asp18	—	Asp18	Asp18	Glu18	Glu18	Glu18	—
	Arg209	Asp54, Asp55, Asp62	Asp55, Gln58, Asp62	Asp54, Asp55	Asp55	Asp54, Asp55	Asp54, Asp55	Asp55, Asp62	—
	Asp210	—	Gln58, Asp62	—	Gln58, Asp62	—	Gln58, Asp62	—	Gln58, Asp62

Salt bridges and hydrogen bonds (H-bonds) were calculated using 100 ns cMD trajectories by VMD1.9.1[8]. The salt bridge was recorded when the distance between one of the carboxyl oxygen atoms of an acidic residue and the nitrogen atom of a

basic residue falls within 3.2 Å for at least one frame. Meanwhile, the hydrogen bond is believed to form when the distance between the donor and receptor is within 3.5 Å and the angle of donor-hydrogen-acceptor is less than 30°. Only the hydrogen bonds present in >10% frames of the trajectories are listed.

Table S6. Pearson correlation coefficients (PCCs) of the functional modes identified in the FMA analysis

ID	System	PCC of FMA	Correlation between functional modes
1	hBBCK	0.690	-0.245
2	BP36L	-0.437	
3	hMMCK	-0.286	-0.320
4	ML36P	0.543	

The correlation between functional modes is defined as the cosine value of the angle between two mode vectors, which ranges from -1 to 1.

Table S7. Comparison of network properties in 10 repeated calculations

Property	hBBCK		BP36L	
	Chain A	Chain B	Chain A	Chain B
Number of nodes	367.0±0.0	363.0±0.0	371.0±0.0	367.3±0.5
Number of edges	952.0±1.6	873.6±2.0	998.4±2.3	953.0±1.5
Degree of residue 36	4±0	2±0	6±0	6±0
Average node degree	5.188±0.009	4.813±0.011	5.382±0.013	5.189±0.008
Average clustering coefficient	0.2114±0.0008	0.1928±0.0012	0.2225±0.0015	0.2221±0.0010

Network properties were calculated by averaging over 10 repeated calculations. In each single repeat, 20% of frames were randomly removed from the trajectories. The sample standard deviation of 0 means that identical values were obtained from all 10 repeats.

Table S8. Residues connected to residue 36 in the networks

Network	hBBCK		BP36L	
	Chain A	Chain B	Chain A	Chain B
Neighbors around residue 36	[20, 38, 39, 40]	[20, 39]	[16, 17, 20, 38, 39, 40]	[16, 17, 20, 38, 39, 40]

The identified connections are robust in the bootstrap test, where 20% of frames were randomly removed from the trajectories and the process was repeated for 10 times.

Table S9. Lengths and numbers of shortest pathways between residue 36 and key interface residues

Key residue pair		hBBCK		BP36L	
		Length	Number	Length	Number
Chain A Pro/Leu36	Glu19	2	1	2	3
	Asp55	3	1	3	2
	Asp62	4	1	4	3
	Arg148	8	57	7	1
	Lys156	9	353	9	432
	Arg209	6	1	6	2
	Asp210	7	1	7	2
Chain B Pro/Leu36	Glu19	2	1	2	3
	Asp55	3	2	3	2
	Asp62	4	1	4	2
	Arg148	7	1	7	6
	Lys156	8	10	8	37
	Arg209	8	6	6	3
	Asp210	8	5	7	8

Table S10. Lengths and numbers of shortest pathways between residue 36 and key interface residues in 10 repeated calculations

Repeat	36-19		36-55		36-62		36-148		36-156		36-209		36-210	
	Length	Number	Length	Number	Length	Number	Length	Number	Length	Number	Length	Number	Length	Number
	hBBCK chain A													
1	2	1	3	1	4	1	8	60	9	374	6	1	7	1
2	2	1	3	1	4	1	8	55	9	351	7	9	8	27
3	2	1	3	1	4	1	8	57	9	353	6	1	7	1
4	2	1	3	1	4	1	8	60	9	374	6	1	7	1
5	2	1	3	1	4	1	8	60	9	374	6	1	7	1
6	2	1	3	1	4	1	8	57	9	353	6	1	7	1
7	2	1	3	1	4	1	8	57	9	353	6	1	7	1
8	2	1	3	1	4	1	8	60	9	374	6	1	7	1
9	2	1	3	1	4	1	8	60	9	374	6	1	7	1
10	2	1	3	1	4	1	8	57	9	353	6	1	7	1
	BP36L chain A													
1	2	3	3	2	4	3	7	1	9	432	6	2	7	2
2	2	3	3	2	4	3	7	1	9	432	6	2	7	2
3	2	3	3	2	4	3	7	1	9	432	6	2	7	2
4	2	3	3	2	4	3	7	1	9	432	6	2	7	2
5	2	3	3	2	4	3	7	1	9	436	6	2	7	2
6	2	3	3	2	4	3	7	1	9	436	6	2	7	2
7	2	3	3	2	4	3	7	1	9	441	6	2	7	2
8	2	3	3	2	4	3	7	1	9	436	6	2	7	2
9	2	3	3	2	4	3	7	1	9	436	6	2	7	2
10	2	3	3	2	4	3	7	1	9	436	6	2	7	2

Repeat	36-19		36-55		36-62		36-148		36-156		36-209		36-210	
	Length	Number	Length	Number	Length	Number	Length	Number	Length	Number	Length	Number	Length	Number
	hBBCK chain B													
1	2	1	3	2	4	1	7	1	8	10	8	6	8	5
2	2	1	3	2	4	1	7	1	8	10	8	6	8	5
3	2	1	3	2	4	1	7	1	8	10	8	5	8	5
4	2	1	3	2	4	1	7	1	8	10	8	6	8	5
5	2	1	3	2	4	1	7	1	8	10	8	5	8	5
6	2	1	3	2	4	1	7	1	8	10	8	5	8	5
7	2	1	3	2	4	1	7	1	8	10	8	6	8	5
8	2	1	3	2	4	1	7	1	8	10	8	5	8	5
9	2	1	3	2	4	1	7	1	8	10	8	5	8	5
10	2	1	3	2	4	1	7	1	8	10	8	5	8	5
	BP36L chain B													
1	2	3	3	2	4	2	7	6	9	440	6	3	7	8
2	2	3	3	2	4	2	7	6	8	37	6	3	7	8
3	2	3	3	2	4	2	7	6	8	37	6	3	7	8
4	2	3	3	2	4	2	7	6	9	440	6	3	7	8
5	2	3	3	2	4	2	7	6	9	440	6	3	7	8
6	2	3	3	2	4	2	7	6	8	37	6	3	7	8
7	2	3	3	2	4	2	7	6	8	37	6	3	7	8
8	2	3	3	2	4	2	7	6	9	440	6	3	7	8
9	2	3	3	2	4	2	7	6	9	440	6	3	7	8
10	2	3	3	2	4	2	7	6	9	440	6	3	7	8

In each repeated calculation, 20% frames of the total trajectory were removed randomly.

Table S11. The cutoff values chosen for clustering the structural snapshots from the aMD trajectories

System	hBBCK	BP36L	hMMCK	ML36P
Cutoff (Å)	2.8	3.0	2.8	2.8
Size of top 8 clusters / all frames	1851/2000	1853/2000	1849/2000	1852/2000

Table S12. Primers designed for the construction of chimeras

Chimera name	Sequence (from 5' to 3')
MnBc-For	GAAAACCTCAAGGGCGGCGACGACCTGGAC
MnBc-Rev	GTCGCCGCCCTTGAGGTTTTCATGGTTGAG
BnMc-For	GACAACCTGCAGGGTGGAGACGACCTGGAC
BnMc-Rev	GTCGTCTCCACCCTGCAGGTTGTCGGGGTTG
$B_{1\text{-}53}$M-For	CTTCACGCTGGACGATGTCATCCAGACAGGAG
$B_{1\text{-}53}$M-Rev	GATGACATCGTCCAGCGTGAAGCCGCTCGGCGTG
$M_{1\text{-}53}$B-For	CTTCACTGTAGACGACGTCATCCAGACAG
$M_{1\text{-}53}$B-Rev	GATGACGTCGTCTACAGTGAAGCCAGATGGAG
$B_{1\text{-}26}$M-For	CACAACAACCACATGGCCAAGG
$B_{1\text{-}26}$M-Rev	CCTTGGCCATGTGGTTGTTGTG
$M_{1\text{-}26}$B-For	CATAACAACCACATGGCCAAGG
$M_{1\text{-}26}$B-Rev	CCTTGGCCATGTGGTTGTTGTG

Table S13. Primers designed for the site-directed mutagenesis

Mutant name	Sequence (from 5' to 3')
Group I	
BP36L-For	CTGACCCTTGAGCTGTACGC
BP36L-Rev	GCGTACAGCTCAAGGGTCAG
BA40K-For	GAGCTGTACAAGGAGCTGCG
BA40K-Rev	CGCAGCTCCTTGTACAGCTC
BE41K-For	GAGCTGTACGCGAAGCTGCG
BE41K-Rev	CGCAGCTTCGCGTACAGCTC
BA44D-For	CTGCGCGACAAGAGCACGCC
BA44D-Rev	GGCGTGCTCTTGTCGCGCAG
BS46E-For	CTGCGCGCCAAGGAGACGCC
BS46E-Rev	GGCGTCTCCTTGGCGCGCAG
BL53V-For	GCTTCACGGTAGACGACGTC
BL53V-Rev	GACGTCGTCTACCGTGAAGC
Group II	
ML36P-For	GGTACTGACCCCCGAACTC
ML36P-Rev	GAGTTCGGGGGTCAGTACC
MK40A-For	GAACTCTACGCGAAGCTGCG
MK40A-Rev	CGCAGCTTCGCGTAGAGTTC
MK41E-For	GAACTCTACAAGGAGCTGCG
MK41E-Rev	CGCAGCTCCTTGTAGAGTTC
MD44A-For	CTGCGGGCCAAGGAGACTCC
MD44A-Rev	GGAGTCTCCTTGGCCCGCAG
ME46S-For	CTGCGGGACAAGAGCACTCC
ME46S-Rev	GGAGTGCTCTTGTCCCGCAG
MV53L-For	GCTTCACTCTGGACGATGTC
MV53L-Rev	GACGTCGTCTACCGTGAAGC

Table S14. Comparison of network properties under various criteria for the network construction

Angle (°)	Distance (Å)	Ratio	Node number	Edge number	Degree of residue 36	Average degree	Average clustering	Node number	Edge number	Degree of residue 36	Average degree	Average clustering
			hBBCK chain A					BP36L chain A				
45	4.5	0.75	367	953	4	5.193	0.212	371	996	6	5.369	0.22
45	4.5	0.65	368	1020	4	5.543	0.222	371	1062	6	5.725	0.225
45	4.5	0.70	367	982	4	5.351	0.216	371	1031	6	5.558	0.231
45	4.5	0.80	366	914	4	4.995	0.202	370	963	6	5.205	0.208
45	4.0	0.75	361	697	4	3.861	0.158	358	743	4	4.151	0.165
45	5.0	0.75	371	1177	5	6.345	0.249	373	1210	7	6.488	0.264
45	5.5	0.75	372	1390	6	7.473	0.309	373	1441	8	7.727	0.318
40	4.5	0.75	365	930	4	5.096	0.211	367	967	6	5.27	0.210
50	4.5	0.75	369	980	4	5.312	0.218	372	1014	6	5.452	0.223
55	4.5	0.75	369	990	4	5.366	0.221	374	1024	6	5.476	0.221
			hBBCK chain B					BP36L chain B				
45	4.5	0.75	363	872	2	4.804	0.192	367	953	6	5.193	0.223
45	4.5	0.65	363	947	2	5.218	0.206	372	1014	6	5.452	0.227
45	4.5	0.70	363	910	2	5.014	0.202	369	984	6	5.333	0.225
45	4.5	0.80	362	835	2	4.613	0.186	366	911	5	4.978	0.207
45	4.0	0.75	358	647	2	3.615	0.146	358	703	4	3.927	0.181
45	5.0	0.75	370	1088	3	5.881	0.228	372	1164	7	6.258	0.257
45	5.5	0.75	371	1295	3	6.981	0.273	372	1367	8	7.349	0.299
40	4.5	0.75	352	832	1	4.727	0.183	364	932	6	5.121	0.223
50	4.5	0.75	363	907	2	4.997	0.197	367	972	6	5.297	0.224
55	4.5	0.75	365	938	2	5.14	0.207	367	997	6	5.433	0.219

Table S15. Comparison on the connections of residue 36 under various criteria for the network construction

Angle (°)	Distance (Å)	Ratio	hBBCK		BP36L	
			Chain A	Chain B	Chain A	Chain B
45	4.5	0.75	[20, 38, 39, 40]	[20, 39]	[16, 17, 20, 38, 39, 40]	[16, 17, 20, 38, 39, 40]
45	4.5	0.65	[20, 38, 39, 40]	[20, 39]	[16, 17, 20, 38, 39, 40]	[16, 17, 20, 38, 39, 40]
45	4.5	0.7	[20, 38, 39, 40]	[20, 39]	[16, 17, 20, 38, 39, 40]	[16, 17, 20, 38, 39, 40]
45	4.5	0.8	[20, 38, 39, 40]	[20, 39]	[16, 17, 20, 38, 39, 40]	[17, 20, 38, 39, 40]
45	4	0.75	[20, 38, 39, 40]	[20, 39]	[20, 38, 39, 40]	[20, 38, 39, 40]
45	5	0.75	[20, 34, 38, 39, 40]	[20, 34, 39]	[16, 17, 20, 34, 38, 39, 40]	[16, 17, 20, 34, 38, 39, 40]
45	5.5	0.75	[20, 34, 38, 39, 40, 41]	[20, 34, 39]	[16, 17, 20, 34, 38, 39, 40, 41]	[16, 17, 20, 34, 38, 39, 40, 41]
40	4.5	0.75	[20, 38, 39, 40]	[20]	[16, 17, 20, 38, 39, 40]	[16, 17, 20, 38, 39, 40]
50	4.5	0.75	[20, 38, 39, 40]	[20, 39]	[16, 17, 20, 38, 39, 40]	[16, 17, 20, 38, 39, 40]
55	4.5	0.75	[20, 38, 39, 40]	[20, 39]	[16, 17, 20, 38, 39, 40]	[16, 17, 20, 38, 39, 40]

Table S16. Comparison of shortest paths between residue 36 and key interface residues under various criteria for network construction

Angle (°)	Distance (Å)	Ratio	36-19		36-55		36-62		36-148		36-156		36-209		36-210	
			Length	Number	Length	Number	Length	Number	Length	Number	Length	Number	Length	Number	Length	Number
			hBBCK chain A													
45	4.5	0.75	2	1	3	1	4	1	8	57	9	353	6	1	7	1
45	4.5	0.65	2	1	3	2	4	2	8	106	8	17	6	3	7	3
45	4.5	0.70	2	1	3	2	4	2	8	82	9	470	6	3	7	3
45	4.5	0.80	2	1	3	1	5	6	8	44	9	286	7	4	8	18
45	4.0	0.75	2	1	3	1	5	1	8	3	9	13	7	1	8	1
45	5.0	0.75	2	1	3	3	4	8	6	1	7	4	6	6	7	13
45	5.5	0.75	2	1	3	5	4	16	6	11	7	91	6	23	7	93
40	4.5	0.75	2	1	3	1	4	1	8	57	9	345	6	1	7	1
50	4.5	0.75	2	1	3	1	4	1	8	57	9	353	6	1	7	1
55	4.5	0.75	2	1	3	1	4	1	8	57	9	353	6	1	7	1
			BP36L chain A													
45	4.5	0.75	2	3	3	2	4	3	7	1	9	432	6	2	7	2
45	4.5	0.65	2	3	3	2	4	3	7	4	8	41	6	2	7	4
45	4.5	0.70	2	3	3	2	4	3	7	4	9	482	6	2	7	2
45	4.5	0.80	2	3	3	2	4	2	7	1	9	365	6	2	7	2
45	4.0	0.75	2	1	3	1	5	4	8	7	9	42	8	8	9	19
45	5.0	0.75	2	3	3	3	4	6	7	35	7	3	6	5	7	12
45	5.5	0.75	2	3	3	4	3	2	6	2	7	59	6	15	7	51
40	4.5	0.75	2	3	3	2	4	3	7	1	9	420	6	2	7	2
50	4.5	0.75	2	3	3	2	4	3	7	1	9	432	6	2	7	2
55	4.5	0.75	2	3	3	2	4	3	7	1	9	432	6	2	7	2

Angle (°)	Distance (Å)	Ratio	36-19		36-55		36-62		36-148		36-156		36-209		36-210	
			Length	Number	Length	Number	Length	Number	Length	Number	Length	Number	Length	Number	Length	Number
			hBBCK chain B													
45	4.5	0.75	2	1	3	2	4	1	7	1	8	10	8	6	8	5
45	4.5	0.65	2	1	3	2	4	1	7	1	8	11	6	1	7	1
45	4.5	0.70	2	1	3	2	4	1	7	1	8	10	6	1	7	1
45	4.5	0.80	2	1	3	1	5	3	7	1	8	7	8	3	8	4
45	4.0	0.75	2	1	3	1	5	1	8	2	9	11	10	5	9	5
45	5.0	0.75	2	1	3	2	4	4	7	20	7	3	6	1	7	2
45	5.5	0.75	2	1	3	2	4	7	7	37	7	13	6	1	7	6
40	4.5	0.75	3	2	4	1	5	2	8	1	9	5	9	2	9	2
50	4.5	0.75	2	1	3	2	4	1	7	1	8	11	8	8	8	5
55	4.5	0.75	2	1	3	2	4	1	7	1	8	19	6	1	7	1
			BP36L chain B													
45	4.5	0.75	2	3	3	2	4	2	7	6	8	37	6	3	7	8
45	4.5	0.65	2	3	3	2	4	3	7	6	8	43	6	3	7	8
45	4.5	0.70	2	3	3	2	4	2	7	6	8	43	6	3	7	8
45	4.5	0.80	2	2	3	2	4	2	7	6	9	435	6	1	7	8
45	4.0	0.75	2	1	3	1	4	1	8	4	9	29	9	27	8	2
45	5.0	0.75	2	3	3	3	4	7	7	37	7	3	6	6	7	18
45	5.5	0.75	2	3	3	4	4	9	6	5	7	58	6	24	7	60
40	4.5	0.75	2	3	3	2	4	2	7	5	8	37	6	3	7	8
50	4.5	0.75	2	3	3	2	4	2	7	6	8	37	6	3	7	8
55	4.5	0.75	2	3	3	2	4	2	7	6	8	37	6	3	7	8

Supplementary references

1 Gao, Y. S. *et al.* Isoenzyme-specific thermostability of human cytosolic creatine kinase. *Int J Biol Macromol* **47**, 27-32, (2010).

2 Bradford, M. M. A rapid and sensitive method for the quantitation of microgram quantities of protein utilizing the principle of protein-dye binding. *Anal Biochem* **72**, 248-254, (1976).

3 Yao, Q. Z., Zhou, H. M., Hou, L. X. & Zou, C. G. A comparison of denaturation and inactivation rates of creatine kinase in guanidine solutions. *Sci Sin B* **25**, 1296-1802, (1982).

4 Zhao, T. J. *et al.* Impact of intra-subunit domain-domain interactions on creatine kinase activity and stability. *FEBS Lett* **580**, 3835-3840, (2006).

5 Eswar, N. *et al.* Comparative protein structure modeling using Modeller. *Curr Protoc Bioinformatics* **Chapter 5**, Unit 5 6, (2006).

6 Sali, A. & Blundell, T. L. Comparative protein modelling by satisfaction of spatial restraints. *J Mol Biol* **234**, 779-815, (1993).

7 Fiser, A., Do, R. K. & Sali, A. Modeling of loops in protein structures. *Protein Sci* **9**, 1753-1773, (2000).

8 Humphrey, W., Dalke, A. & Schulten, K. VMD: visual molecular dynamics. *J Mol Graph* **14**, 33-38, 27-38, (1996).

9 Salomon-Ferrer, R., Case, D. A. & Walker, R. C. An overview of the Amber biomolecular simulation package. *Wiley Interdisciplinary Reviews: Computational Molecular Science* **3**, 198-210, (2013).

10 Joung, I. S. & Cheatham, T. E., 3rd. Determination of alkali and halide monovalent ion parameters for use in explicitly solvated biomolecular simulations. *J Phys Chem B* **112**, 9020-9041, (2008).

11 Hamelberg, D., Mongan, J. & McCammon, J. A. Accelerated molecular dynamics: a promising and efficient simulation method for biomolecules. *J Chem Phys* **120**, 11919-11929, (2004).

12 Wang, Y., Harrison, C. B., Schulten, K. & McCammon, J. A. Implementation of Accelerated Molecular Dynamics in NAMD. *Comput Sci Discov* **4**, (2011).

13 Phillips, J. C. *et al.* Scalable molecular dynamics with NAMD. *J Comput Chem* **26**, 1781-1802, (2005).

14 Feller, S. E., Zhang, Y., Pastor, R. W. & Brooks, B. R. Constant pressure molecular dynamics simulation: The Langevin piston method. *The Journal of Chemical Physics* **103**, 4613-4621, (1995).

15 Berendsen, H. J. C., Postma, J. P. M., van Gunsteren, W. F., DiNola, A. & Haak, J. R. Molecular dynamics with coupling to an external bath. *The Journal of Chemical Physics* **81**, 3684-3690, (1984).

16 Essmann, U. *et al.* A smooth particle mesh Ewald method. *The Journal of Chemical Physics* **103**, 8577-8593, (1995).

17 Miyamoto, S. & Kollman, P. Settle: An analytical version of the SHAKE and RATTLE algorithm for rigid water models. *Journal of Computational Chemistry* **13**, 952-962, (1992).

18 Hamelberg, D., de Oliveira, C. A. & McCammon, J. A. Sampling of slow diffusive conformational transitions with accelerated molecular dynamics. *J Chem Phys* **127**, 155102, (2007).

19 Wereszczynski, J. & McCammon, J. A. Accelerated molecular dynamics in computational drug design. *Methods Mol Biol* **819**, 515-524, (2012).
20 Wereszczynski, J. & McCammon, J. A. Nucleotide-dependent mechanism of Get3 as elucidated from free energy calculations. *Proc Natl Acad Sci U S A* **109**, 7759-7764, (2012).
21 Case, D. A. *et al. AMBER 12*. (University of California, 2012).
22 Luo, R., David, L. & Gilson, M. K. Accelerated Poisson-Boltzmann calculations for static and dynamic systems. *J Comput Chem* **23**, 1244-1253, (2002).
23 Tan, C., Yang, L. & Luo, R. How Well Does Poisson-Boltzmann Implicit Solvent Agree with Explicit Solvent? A Quantitative Analysis. *J Phys Chem B* **110**, 18680-18687, (2006).
24 Hub, J. S. & de Groot, B. L. Detection of functional modes in protein dynamics. *PLoS Comput Biol* **5**, e1000480, (2009).
25 Krivobokova, T., Briones, R., Hub, J. S., Munk, A. & de Groot, B. L. Partial least-squares functional mode analysis: application to the membrane proteins AQP1, Aqy1, and CLC-ec1. *Biophys J* **103**, 786-796, (2012).
26 Van Der Spoel, D. *et al.* GROMACS: fast, flexible, and free. *J Comput Chem* **26**, 1701-1718, (2005).
27 Bakan, A., Meireles, L. M. & Bahar, I. ProDy: protein dynamics inferred from theory and experiments. *Bioinformatics* **27**, 1575-1577, (2011).
28 Sethi, A., Eargle, J., Black, A. A. & Luthey-Schulten, Z. Dynamical networks in tRNA:protein complexes. *Proc Natl Acad Sci U S A* **106**, 6620-6625, (2009).
29 Ermakova, E. & Kurbanov, R. Effect of ligand binding on the dynamics of trypsin. Comparison of different approaches. *J Mol Graph Model* **49**, 99-109, (2014).
30 Vanwart, A. T., Eargle, J., Luthey-Schulten, Z. & Amaro, R. E. Exploring residue component contributions to dynamical network models of allostery. *J Chem Theory Comput* **8**, 2949-2961, (2012).
31 Hagberg, A. A., Schult, D. A. & Swart, P. J. Exploring Network Structure, Dynamics, and Function using NetworkX. *Proceedings of the 7th Python in Science Conference*, 11-15, (2008).
32 Schank, T. & Wagner, D. Approximating Clustering Coefficient and Transitivity. *Journal of Graph Algorithms and Applications* **9**, 265-275, (2005).
33 Balasubramaniam, D. *et al.* How the ankyrin and SOCS box protein, ASB9, binds to creatine kinase. *Biochemistry* **54**, 1673-1680, (2015).
34 Smart, O. S., Goodfellow, J. M. & Wallace, B. A. The pore dimensions of gramicidin A. *Biophys J* **65**, 2455-2460, (1993).
35 Smart, O. S., Neduvelil, J. G., Wang, X., Wallace, B. A. & Sansom, M. S. HOLE: a program for the analysis of the pore dimensions of ion channel structural models. *J Mol Graph* **14**, 354-360, 376, (1996).
36 Weiner, S. J. *et al.* A new force field for molecular mechanical simulation of nucleic acids and proteins. *J Am Chem Soc* **106**, 765-784, (1984).
